# Unsaturated and Benzannulated *N*-Heterocyclic Carbene Complexes of Titanium and Hafnium: Impact on Catalysts Structure and Performance in Copolymerization of Cyclohexene Oxide with CO_2_

**DOI:** 10.3390/molecules25194364

**Published:** 2020-09-23

**Authors:** Lakshmi Suresh, Ralte Lalrempuia, Jonas B. Ekeli, Francis Gillis-D’Hamers, Karl W. Törnroos, Vidar R. Jensen, Erwan Le Roux

**Affiliations:** 1 Department of Chemistry, University of Bergen, Allégaten 41, N-5007 Bergen, Norway; Lakshmi.Suresh@uib.no (L.S.); lalrempuia.ralte@dcu.ie (R.L.); Jonas.Ekeli@student.uib.no (J.B.E.); Francis.gillis567@gmail.com (F.G.-D.); Karl.Tornroos@uib.no (K.W.T.); 2 School of Chemical Sciences, Dublin City University, Dublin 9, Ireland

**Keywords:** *N*-heterocyclic carbene, titanium, hafnium, copolymerization of epoxide with CO_2_, density functional theory, natural bond orbitals

## Abstract

Tridentate, bis-phenolate *N*-heterocyclic carbenes (NHCs) are among the ligands giving the most selective and active group 4-based catalysts for the copolymerization of cyclohexene oxide (CHO) with CO_2_. In particular, ligands based on imidazolidin-2-ylidene (saturated NHC) moieties have given catalysts which exclusively form polycarbonate in moderate-to-high yields even under low CO_2_ pressure and at low copolymerization temperatures. Here, to evaluate the influence of the NHC moiety on the molecular structure of the catalyst and its performance in copolymerization, we extend this chemistry by synthesizing and characterizing titanium complexes bearing tridentate bis-phenolate imidazol-2-ylidene (unsaturated NHC) and benzimidazol-2-ylidene (benzannulated NHC) ligands. The electronic properties of the ligands and the nature of their bonds to titanium are studied using density functional theory (DFT) and natural bond orbital (NBO) analysis. The metal–NHC bond distances and bond strengths are governed by ligand-to-metal σ- and π-donation, whereas back-donation directly from the metal to the NHC ligand seems to be less important. The NHC π-acceptor orbitals are still involved in bonding, as they interact with THF and isopropoxide oxygen lone-pair donor orbitals. The new complexes are, when combined with [PPN]Cl co-catalyst, selective in polycarbonate formation. The highest activity, albeit lower than that of the previously reported Ti catalysts based on saturated NHC, was obtained with the benzannulated NHC-Ti catalyst. Attempts to synthesize unsaturated and benzannulated NHC analogues based on Hf invariably led, as in earlier work with Zr, to a mixture of products that include zwitterionic and homoleptic complexes. However, the benzannulated NHC-Hf complexes were obtained as the major products, allowing for isolation. Although these complexes selectively form polycarbonate, their catalytic performance is inferior to that of analogues based on saturated NHC.

## 1. Introduction

In the past few decades, *N*-heterocyclic carbenes (NHCs) have emerged as privileged ancillary ligands that, in particular, have been explored in combination with low-to-medium valent late-transition-metals due to their strong σ-donor capacity, structural diversity, and their successful use in organometallic catalysis [1,2,3,4,5,6,7,8]. In contrast, high-valent early-transition-metal NHC complexes have received much less attention [9,10,11,12,13,14,15], which to a large extent is due to their ease of dissociation from these metal centers [2,5,9,11,12,14]. However, their dissociation from oxophilic metals was partially prevented by designing multidentate anionic carbon, nitrogen, and oxygen-functionalized NHC ligands [5,13,15,16,17]. Anchoring such functionalized NHC ligands to oxophilic metals has proved to be a successful approach for developing catalysts, notably of group 4 metals, for the (oligo-)polymerization of olefins [18,19,20,21,22,23,24,25,26,27,28,29], hydroamination/cyclization of aminoalkenes [30,31,32,33,34,35,36], controlled ring-opening polymerization of *rac*-lactide [37,38,39,40], and more recently, for the copolymerization of epoxides with CO_2_ [41,42,43,44,45].

**Scheme 1:**
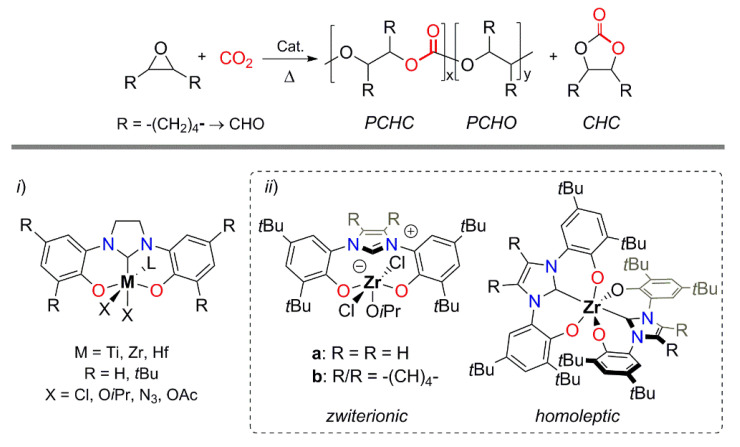
Previously obtained complexes containing (***i***) saturated NHC with group 4, and (***ii***) unsaturated and benzannulated NHCs ligands (**a** and **b**, respectively) with zirconium.

Although most of the highly selective and active catalyst systems for the copolymerization of epoxides with CO_2_ are based on divalent (Mg, Co, Zn) and trivalent (Cr, Co, Al) metals bearing ligands such as β-diketiminates, salens, porphyrins, and multidentate phenolate macrocycles [46,47,48,49,50,51,52], the group 4 metals have emerged as a new class of catalyst for this reaction, combining decent catalytic activity with high selectivity towards the formation of polycarbonates [41,42,43,44,45,53,54,55,56,57,58,59,60,61,62,63,64,65,66]. NHC-based catalysts of this kind have appeared to be particularly promising under relatively mild copolymerization conditions [41,42,43,44,45]. However, so far the tetravalent titanium, zirconium and hafnium complexes for the CO_2_/cyclohexene oxide (CHO) copolymerization that have selectively given the desired poly(cyclohexene carbonate) (PCHC), without cyclohexene carbonate (CHC) or homopolymer of CHO (PCHO) as side products, have exclusively been based on tridentate bis-phenolate NHC-type ligands with saturated backbones (Scheme 1) [41,42,43,44,45]. Previous attempts to substitute the saturated-NHC backbone of zirconium catalysts by unsaturated- or benzannulated-NHC backbones in order to evaluate the effects on the copolymerization catalysis have irremediably led to a catalytically inactive isolable mixture of zwitterionic and heteroleptic zirconium compounds (Scheme 1) [44]. This suggests that strong σ-donation from the NHC ligand to the metal is vital for CO_2_/CHO copolymerization.

We have continued our efforts to synthesize catalysts based on unsaturated NHC ligands, and present here, for the first time, bis-phenolate unsaturated- and benzannulated-NHC complexes of titanium and hafnium. The performance of these complexes in CO_2_/CHO copolymerization is compared to those of saturated NHC ligands. Finally, with the help of density functional theory (DFT) and natural bond orbital (NBO) analysis, the structural and electronic property differences of these complexes are presented, and the potential impact of these differences on CO_2_/CHO copolymerization is discussed.

## 2. Results and Discussion

### 2.1. Synthesis of Bis-Phenolate NHC Complexes of Titanium and Hafnium

**Scheme 2:**
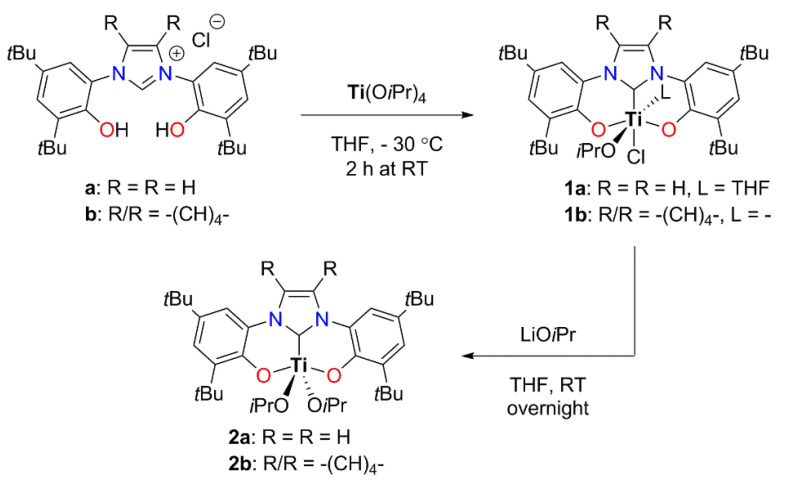
Synthesis of NHC-Ti complexes **1a**, **1b**, **2a** and **2b**.

The *N*,*N*′-di(2-hydroxy-3,5-di-*tert*-butylphenyl) imidazolium chloride (**a**) and *N*,*N*′-di(2-hydroxy-3,5-di-*tert*-butylphenyl) benzoimidazolium chloride (**b**) proligands were prepared according to previously reported procedures [26,67,68]. The alcohol elimination route involving the direct and slow addition of proligands **a** and **b** to a solution of Ti(O*i*Pr)_4_ in THF at −30 °C was found to be the most appropriate protocol for the synthesis of both ([*κ*^3^*-*O,C,O]-^I^NHC)TiCl(O*i*Pr)(THF) **1a** and ([*κ*^3^*-*O,C,O]-^Bz^NHC)TiCl(O*i*Pr) **1b** complexes, respectively, in good yields and without the formation of side compounds, such as the homoleptic and zwitterionic complexes (Scheme 2). This protocol slightly diverges from the previously reported one in which *N*,*N*′-di(2-hydroxy-3,5-di-*tert*-butylphenyl) imidazolidinium chloride salt (**c**) was used as proligand and the addition was carried out at room temperature, leading quantitatively to ([*κ*^3^*-*O,C,O]-^Is^NHC)TiCl(O*i*Pr)(THF) **1c** [38].

The ^1^H and ^13^C-NMR spectra show that the proligands are fully deprotonated with the concomitant disappearance of both OH and H_imidazolium_ protons and all chemical resonances are shifted downfield in agreement with the bonding of bis-phenolate NHC ligands to the titanium metal center for both **1a** and **1b** compounds (Supplementary Figures S1–S4). The only immediately observed difference between these two compounds is that the THF molecule in complex **1b** is very labile and can easily be removed under prolonged vacuum. The ^13^C-NMR spectra of both compounds **1a** and **1b** encompass typical imidazol-2-ylidene and benzimidazolin-2-ylidene NHC-C_carbene_ resonances at *δ* 184.0 and 195.2 ppm (Supplementary Figures S2 and S4) [14,69], respectively, which are shifted upfield compared to that of the structurally analogous bis-phenolate saturated NHC complex of titanium ([*κ*^3^*-*O,C,O]-^Is^NHC)TiCl(O*i*Pr)(THF) **1c** (*δ* 198.6 ppm) [38].

As indicated above, the order of addition of the reagents is crucial here, contrasting the case of the Zr(O*i*Pr)_4_(HO*i*Pr) precursor with either **a** or **b** proligands in which the homoleptic and zwitterionic compounds are observed independently of the addition order [44]. For instance, the addition of Ti(O*i*Pr)_4_ to proligand **b** in THF at room temperature leads to a mixture of compounds containing at least complex **1b** (unambiguously deduced from by ^1^H-NMR analysis, cf. Figure S5) along with the zwitterionic ([κ^2^-O,O]-^Bz^NHC-H)TiCl_2_(O*i*Pr) **1b’** and homoleptic ([*κ*^3^*-*O,C,O]-^Bz^NHC)_2_Ti **1b’’** as minor products (9% and 5%, respectively). Consistent with the observations made earlier for the ^Bz^NHC-Zr analogue [44], formation of zwitterionic **1b’** was further confirmed by single-crystal X-ray diffraction (SCXRD) analysis of a crystal sampled from the reaction mixture in toluene at −30 °C (Supplementary Figure S6 and Table S1).

**Figure 1:**
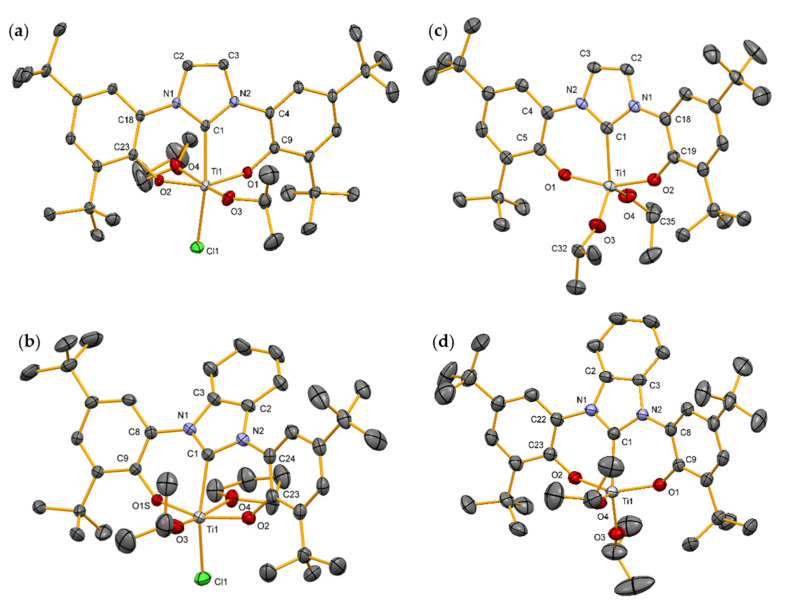
Molecular structures of (**a**) **1a**, (**b**) **1b-THF** (isomer A), (**c**) **2a** and (**d**) **2b**. Hydrogen atoms and solvent molecules are omitted for clarity. Anisotropic displacement parameters (ADP’s) are given at the 50%pobability level.

Similarly, the molecular structures of **1a** and the THF-adduct of **1b** (**1b**-**THF**) were both confirmed by SCXRD analysis (Figure 1a,b). Selected bond lengths, angles and torsion angles are shown in Table 1. Further crystallographic information and data for **1a** and **1b-THF** are given in Table S2. 

Both **1a** and **1b-THF** show a slightly distorted octahedral geometry around the Ti(IV) center as a result of the *mer*-coordination of the tridentate NHC ligand, with ∠O_Ar_-Ti-O_Ar_ bite angles of 159.14(4)° and 159.18(8)°, respectively. This ligand coordination is similar to that observed earlier for other bis-phenolate NHC-Ti complexes [20,29,38,40,42,70].

The principal structural features of both **1a** and **1b-THF** include (i) a *mer*-NHC chelate deviating from planarity, with torsion angles ∠O_Ar_-C_Ar_-N-C_carbene_ of 5.86/6.89° and 27.04/–25.40°, respectively, (ii) *trans*-dispositioning of the carbene moiety and the Cl atom, and (iii) *trans*-dispositioning of the O*i*Pr and the THF ligand. Compared to the corresponding titanium complex bearing a saturated NHC, ([*κ*^3^*-*O,C,O]-^Is^NHC)TiCl(O*i*Pr)(THF) **1c**, the torsion angles ∠O_Ar_-C_Ar_-N-C_carbene_ are similar for **1a** but far more distorted from planarity for **1b-THF** (Table 1) [38]. The Ti–C_carbene_ bond length (2.221(2) Å) in complex **1b-THF** is longer than that of 1c (2.166(3) Å), reflected in a shorter Ti-Cl bond distance (2.3459(8) Å) *trans* to the NHC. This is consistent with a weaker *trans* influence from the presumably less electron-donating benzimidazolin-2-ylidene moiety [2,71,72,73]. Less electron donation from the latter ligand and its deviation from planarity might help explain the relatively short Ti-O*i*Pr bond and the tendency toward *sp*^2^ hybridization, suggested by the relatively wide ∠Ti-O-C*_i_*_Pr_ angle (160.5(3)°, 165.57(19)° for isomer B), for this oxygen atom. The more *sp*^2^-like hybridization may bring about increased π-donation from the O*i*Pr moiety and thus explain the apparent greater *trans* influence and the more weakly bound THF molecule in **1b-THF** (Ti-THF_avg_ ≈ 2.28 Å) and **1c** than in **1a** (Table 1).

The latter complex has the shortest Ti–C_carbene_ bond distance (2.1310(13) Å) of all reported NHC-Ti complexes of functionalized NHC ligands (Ti-C_carbene_ = 2.14–2.33 Å) [12,14,29]. As expected, the short Ti–C_carbene_ bond is, due to *trans* influence, reflected in a Ti–Cl*_trans_* bond that is longer than in **1b-THF** and only slightly shorter than in **1c**. Whereas ligands based on the imidazolidin-2-ylidene moiety are often reported to be more electron donating than those of the imidazol-2-ylidene moiety [2,71,72,73], the short Ti-C_carbene_ bond distance of **1a** seems to suggest otherwise. The components of the Ti-C_carbene_ bonds of the three ligands have thus been studied and compared using DFT and NBO analysis (vide infra). Furthermore, the relatively sharp ∠Ti-O-C*_i_*_Pr_ angle (139.95(5)°) of **1a** seems to suggest more *sp*^3^*-*like hybridization and less π-donation of the O*i*Pr ligand. The sharp ∠Ti-O-C*_i_*_Pr_ angle appears not to be caused by steric repulsion between the imidazol-2-ylidene and O*i*Pr moieties, since the NHC in **1a** is only slightly less planar than that in **1c**. Thus, the presumed weaker π-donation from O*i*Pr in **1a** is consistent with the short Ti-THF bond (2.2573(11) Å) which, in turn, is consistent with the *trans* influence of O*i*Pr being weaker in **1a** than in **1b-THF** and **1c**.

To further investigate the structural differences of the complexes and their relation to the electronic properties of the NHC ligands, we studied the ligands and the complexes using DFT and NBO analyses. First, the DFT calculations predict the experimentally obtained bond distances accurately, to within 0.01–0.02 Å (Table 1). More importantly, the trend in calculated Ti-C_carbene_ bond distances between the complexes faithfully reproduces that obtained in X-ray crystallographic analysis. The large variation in Ti-C_carbene_ bond distances (up to 9 pm when comparing the X-ray structures) are thus not the result of crystal-packing effects but must instead originate from the carbenes themselves. The experimentally and computationally obtained Ti-C_carbene_ distances thus suggest that the strength of the interaction between the metal and the carbene diminishes in the order **a** > **c** > **b** for the three NHC ligands (Table 1). Valence and torsional angles are also well reproduced, except for the ∠Ti-O-C*_i_*_Pr_ angle. However, this angle varies by more than 20° between the three complexes, presumably reflecting a very shallow bending potential.

Regarding the Ti–ligand bond energies and interactions, the bond “snapping” energies (Table 2), i.e., the bond energies calculated by dissociating the tridentate ligands heterolytically to frozen-geometry [TiCl(O*i*Pr)(THF)]^2+^ fragments **M1a–c** and dianionic NHC ligands **Ma–c** (Supplementary Scheme S1), are consistent with the trend in Ti-C_carbene_ bond distances. Orbital interactions between these pairs of fragments might thus reveal the origin of the trends in both Ti-C_carbene_ bond distances and bond energies. The calculated ligand-to-metal net electron donations (Table 2) are essentially identical for the three complexes, showing that further resolution is necessary for uncovering the factors determining the differences in bond distances and energies.

**Figure 2:**
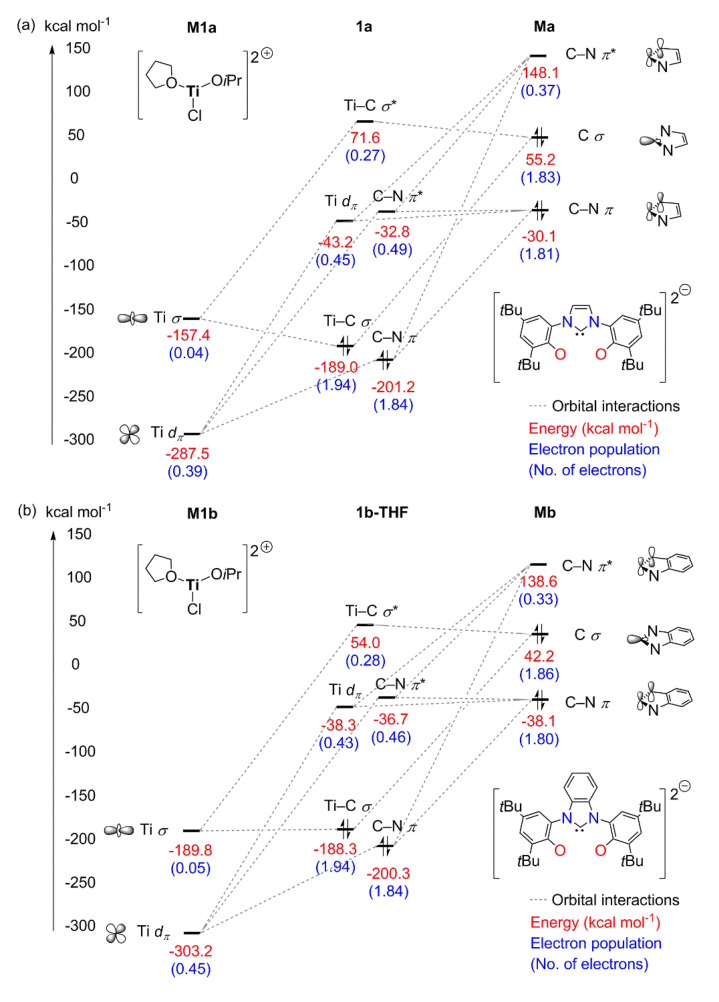
(**a**) Natural orbitals calculated for the **M1a** and **Ma** fragments and the **1a** complex. (**b**) Natural orbitals calculated for the **M1b** and **Mb** fragments and the **1b-THF** complex. The interactions between fragment orbitals leading to hybrid, bonding, or antibonding orbitals of the complexes are indicated by dashed lines.

**Figure 3:**
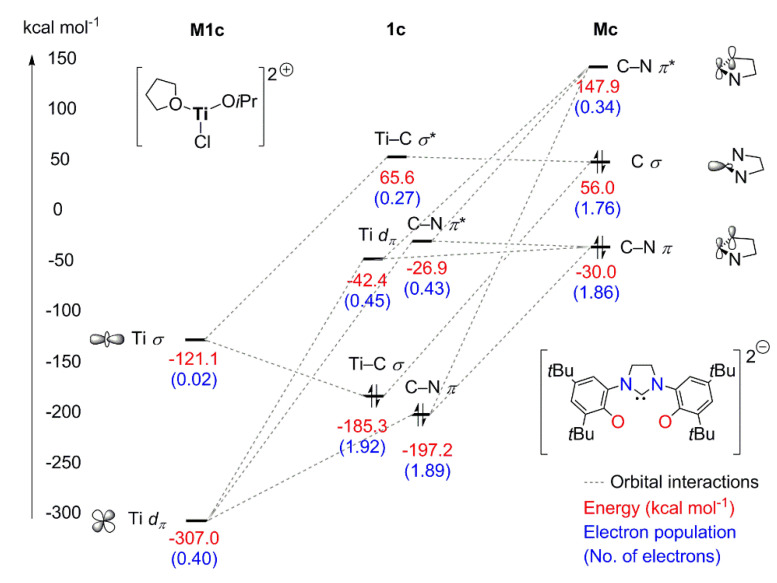
Natural orbitals calculated for the **M1c** and **Mc** fragments and the **1c** complex. The interactions between fragment orbitals leading to hybrid, bonding, or antibonding orbitals of the complex are indicated by dashed lines.

To uncover these factors, we performed NBO [74] analyses of the individual fragments **M1a–c** and **Ma–c** as well as of the metal-ligand orbital interactions in the three complexes. The most important fragment and complex orbitals obtained in these analyses are shown in Figure 2 and Figure 3. 

Insight is offered, for example, by the calculated energies of the C σ orbitals of the three OH-containing free-carbene ligands **Ma–cH_2_** (Table 3 and Supplementary Scheme S1), which suggest that the σ-donating capacity should be greatest for **c**, followed by **a** and **b**. Whereas this ranking is consistent with the relative Ti-C_carbene_ bond distances of **1a** and **1b-THF**, additional factors must explain why this distance is shorter in **1a** than in **1c**. An explanation might be offered by the interaction between the π-orbital of the ligand (C-N π) and a metal *d*-orbital of the same symmetry (Ti *d*_π_). 

The calculated second-order perturbative estimate of the donor-acceptor interaction between these two orbitals is largest for **1a**, followed by **1c**, and **1b-THF** (Table 4 and Supplementary Table S4), consistent with the trend in bond distances and bond energies. In other words, π-donation from the ligand to the metal is suggested to be stronger for **a** and to modify the trend offered by the ligand σ-donating capacity suggested by the C σ energies in Table 3. The importance of ligand-to-metal π-donation has already been noted for NHC complexes of early transition metals [75,76,77,78,79].

Whereas the above-described donation from largely filled ligand π-orbitals to largely empty d_π_-orbitals of the metal is estimated to contribute significantly to the Ti-NHC bonding, the low occupations of metal *d*-orbitals of early transition-metal complexes (see, e.g., Figure 2 and Figure 3) suggest that π-back donation from titanium to the NHC is much less important for the present complexes than for complexes of mid-to-late transition metals [71,75]. The metal *d*-orbitals are considered to be “lone vacant” orbitals in the NBO analysis (Figure 2 and Figure 3), and direct back-donation from the metal to the C-N π* orbitals does not appear in the analysis and is likely to be small. 

In contrast, the C-N π* orbitals are reported to sometimes accept electrons from lone pairs of anionic ligands of early transition metals [76,77,78,79]. Weak contributions of this kind, between isopropoxide oxygen lone pairs and the C-N π* orbitals, are seen also in the present three complexes (Table 4). In addition, the second-order perturbation analysis also identifies analogous interactions between the THF oxygen lone pairs and C-N π*. The strongest of these interactions is in **1b-THF**, where it is likely to be one of the driving forces behind the tilting of the NHC ligand toward the THF.

In conclusion, the calculations show that the strength of the interactions between the metal and the NHC follows the trend portrayed by the calculated and experimental Ti-NHC bond distances (**a** > **c** > **b**). Although the ligand-to-metal σ-donation is predicted to be stronger for ligand **c** (followed by **a**, and **b**), the π-donation from **a** is stronger and contributes to giving the overall trend in metal-ligand interaction strength and bond distances. Whereas back-donation from the metal to the NHC seems to be unimportant, weak donor-acceptor interactions from THF and O*i*Pr lone pairs to the C-N π* orbitals contribute and are probably involved in the tilting of the NHC seen in **1b-THF**.

Due to their potential application in polymerization of CHO with CO_2_ [29,41,42,43], the bis-isopropoxide ^I^NHC- and ^Bz^NHC-titanium complexes **2a** and **2b** were also synthesized and were found to be readily accessible, in quantitative yields (Scheme 2), via salt metathesis of LiO*i*Pr with complex **1a** and **1b**, respectively, similarly to the saturated NHC-titanium analogue [38].

The NMR spectra of **2a** and **2b** contain resonances typical of five-coordinate ([*κ*^3^*-*O,C,O]- NHC)TiX_2_ complexes including a doublet resonance originating from the Me groups of the two O*i*Pr moieties (Supplementary Figures S7–S10), which are consistent with *C*_2v_-symmetric structures in solution for both complexes [38,42,43]. The corresponding ^13^C NMR spectra confirm the chelation of the NHC ligand to Ti center with typical chemical resonances at *δ* 183.7 and 198.1 ppm (Supplementary Figures S8 and S10) [14,69], respectively, shifted upfield compared to the saturated ([*κ*^3^*-*O,C,O]-^Is^NHC)Ti(O*i*Pr)_2_ complex **2c** [38]. Furthermore, the complete molecular structures of **2a** and **2b** were confirmed by SCXRD analysis, showing that these complexes are five-coordinate and adopt a distorted square-pyramidal geometry according to the Addison and Reedijk geometric parameter (*τ*_5_ = 0.49 for **2a** and 0.27 for **2b**), with one of the O*i*Pr moieties in apical position (Figure 1; see Supplementary Table S6 for crystallographic data) [80]. Both geometries differ from that of saturated ([*κ*^3^*-*O,C,O]-^Is^NHC)Ti(O*i*Pr)_2_ complex **2c** in which the five-coordinate Ti metal center adopt a trigonal-bipyramidal geometry (*τ*_5_ = 0.51) [38]. The overall structural data for **2a** and **2b** resemble those previously observed for **1a** and **1b-THF**, with the following main particularities: (i) an even more pronounced deviation from planarity for the *mer*-NHC chelate, with torsion angles ∠O_Ar_-C_Ar_-N-C_carbene_ of −12.88/19.19° for **2a** and −28.95/30.36° for **2b**, (ii) a shorter Ti–C_carbene_ bond length for **2a** compared to **2b** and to the saturated ([*κ*^3^*-*O,C,O]-^Is^NHC)Ti(O*i*Pr)_2_ complex, and (iii) ∠Ti-O-C*_i_*_Pr_ angles approaching linearity (162.7(3) for **2a** and 158.0(3)° for **2b**) for the O*i*Pr co-ligand, indicating enhanced π-donation from this ligand for **2a** compared to **2b** and to the saturated-NHC analogue **2c** (Supplementary Table S6) [38]. The sharper ∠O_Ar_-Ti-O_Ar_ angle (138.19(8)°) observed for **2b** compared to **1b-THF** is most likely a result of steric interactions between the *t*Bu and O*i*Pr moieties (Supplementary Table S6). 

**Scheme 3:**
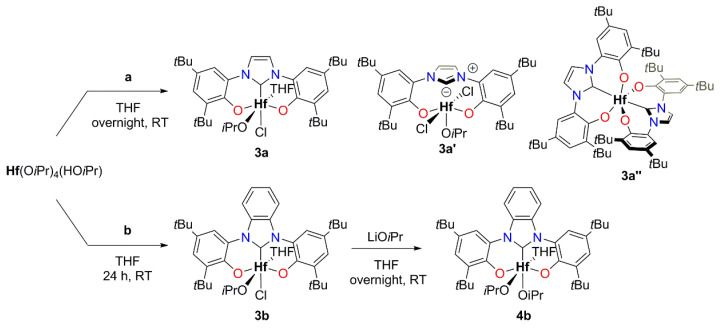
Preparation of NHC-Hf complexes **3a**, **3a’**, **3a’’**, **3b** and **4b**.

Aiming to further explore NHC-hafnium compounds as precursors for the copolymerization of epoxide with CO_2_, attempts to synthesize ([*κ*^3^*-*O,C,O]-^I^NHC)HfCl(O*i*Pr)(THF) **3a** complex via addition of proligand **a** to Hf(O*i*Pr)_4_(HO*i*Pr) under the same reaction conditions as for titanium, invariably gave a mixture of unidentifiable compounds. Only when the addition of **a** to Hf(O*i*Pr)_4_(HO*i*Pr) was performed overnight at room temperature and extended reaction time did the ^1^H NMR spectrum of the reaction mixture showed three distinct sets of signals attributable to three different compounds (in ratio ≈ 2:1:0.8), which unfortunately could not be further separated or isolated. The most intense signal set was tentatively attributed to ([*κ*^3^*-*O,C,O]-^I^NHC)HfCl(O*i*Pr)(THF) **3a**, and the two others to the zwitterionic ([κ^2^-O,O]-^Bz^NHCH)HfCl_2_(O*i*Pr) **3b’** and the homoleptic ([*κ*^3^*-*O,C,O]-^I^NHC)_2_Hf **3a’’** (Scheme 3). 

**Figure 4:**
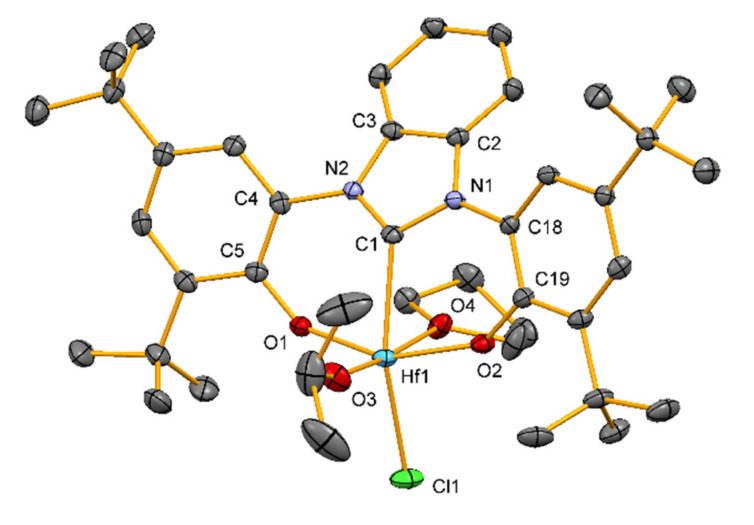
Molecular structure of **3b**. Hydrogen atoms and pentane solvent molecule are omitted for clarity. ADP’s are given at the 50% probability level.

Similarly, addition of proligand **b** to Hf(O*i*Pr)_4_(HO*i*Pr) under identical conditions led to a mixture of compounds, among which ([*κ*^3^*-*O,C,O]-^Bz^NHC)Hf(O*i*Pr)(THF) **3b** is identified to be the major product according to ^1^H NMR (estimated yield 86%, Supplementary Figure S11) and ^13^C-NMR spectra (with a typical Hf-C_carbene_ at *δ* 201.8 ppm) [14,69]. The minor side-products presumably are the zwitterionic **3b’** and the homoleptic **3b’’** (Scheme 3). As previously observed for the reactivity of proligands **a** and **b** with the Zr-alkoxide precursor, the formation of the homoleptic complex cannot be completely avoided, most likely due to the reaction of a second proligand with the large metal ions such as Hf^4+^. In contrast, the smaller Ti^4+^ leads to release of HCl, which, in turn, cleaves off the M-C_carbene_ bond in the ([*κ*^3^*-*O,C,O]-NHC)MCl(O*i*Pr)(THF) complex and thus to the formation zwitterionic species [44]. Even if **3b** could not be further purified, the molecular structure was established by the recovery of single crystals of **3b** suitable for SCXRD analysis from a solution of unpurified **3b** in pentane at −30 °C (Figure 4). A crystallographic summary for **3b** is included with the selected bond lengths, angles, and torsion angles in the electronic supplementary information (Tables S5 and S7). As expected, complex **3b** exhibits structural features closely related to those of **1b-THF**. Similar observations can be made when **3b** is structurally compared to its saturated analogue ([*κ*^3^*-*O,C,O]-^Is^NHC)HfCl(O*i*Pr)(THF) **3c** than between NHC-Ti complexes of **1b-THF** and **1c** [45]. The only exception is the angle ∠Hf-O-C*_i_*_Pr_, which is sharper in the case of **3b** than in **3c** (162.4(3)° vs. 171.1(3)°), indicating slightly diminished π-donation from the O*i*Pr moiety.

As previously reported for the bis-isopropoxide ^Bz^NHC-titanium complex **2b**, the Hf analogue **4b** was also synthesized via salt metathesis from the reaction of LiO*i*Pr with complex **3b** (Scheme 3). Although many attempts to isolate the **4b** in its pure form were unsuccessful, the NMR data unambiguously allowed identification of **4b** as the major product (Supplementary Figure S12).

### 2.2. Copolymerization of CHO with CO_2_

The copolymerization of CHO and CO_2_ was investigated by using unsaturated and benzannulated NHC-titanium and hafnium complexes in combination with 1 equiv. of bis(triphenylphosphine)iminium chloride ([PPN]Cl) as ionic co-catalyst in neat CHO (CHO:M = 1250:1) under mild conditions (*P*_CO2_ = 2 bar, at 65 °C) for 24 h (Table 5). 

The results were compared with the benchmark saturated-NHC complexes ([*κ*^3^*-*O,C,O]-^Is^NHC)TiCl(O*i*Pr)(THF) **1c** and ([*κ*^3^*-*O,C,O]-^Is^NHC)HfCl(O*i*Pr)(THF) **3c** (Table 5) [41,42,43,45]. As for the benchmark binary catalyst systems, all NHC-Ti and NHC-Hf catalysts gave completely alternating PCHC selectively (99% in carbonate linkage) without concomitant formation of CHC or PCHO. Another characteristic feature of the new catalysts was that they all produced PCHCs of molecular weights below 4.5 kg mol^−1^, with bimodal distributions and relatively narrow polydispersities (*Ɖ* < 1.6), indicating a controlled polymerization (entries 1−5 and 7−8, Table 5). A noticeable trend among the NHC-Ti catalysts is that the unsaturated NHC-Ti **1−2a**/[PPN]Cl systems are less active and productive than the benzannulated NHC-Ti **1−2b**/[PPN]Cl systems (entries 1−3 and 5, Table 5). To allow for a direct comparison with the benchmark saturated NHC-Ti **1c** catalyst, the reaction time was shortened to 5 h for avoiding recurrent mass transfer issues about half conversion in neat CHO [41]. It was found that saturated catalyst system **5**/[PPN]Cl is twice as active as the benzannulated NHC-Ti **1b**/[PPN]Cl system (entries 4 and 6, TOFs 62 h^−1^ vs. 116 h^−1^, respectively). Even more pronounced differences in activity were observed when comparing the benzannulated NHC-Hf **3b** and **4b**/[PPN]Cl and the saturated NHC-Hf **3c** (entries 7−9; TOFs 6−7 h^−1^ vs. 116 h^−1^). 

The trend in catalytic activity (**1c** > **1b-THF** > **1a**) might originate from the inherent stability of the complexes and might also, at least in part, originate from the lability of the THF molecule and the ease with which this ligand is displaced to form the putative anionic six-coordinate intermediate upon activation with [PPN]Cl, as previously shown with the anionic catalysts [([*κ*^3^*-*O,C,O]-^Is^NHC)HfCl_3_]^−^ [45] and [([*κ*^4^*-*N,O,O,O]-ATP)TiCl(O*i*Pr)]^−^ (ATP = amino-tris(phenolate)) [62]. The more active catalysts **1c** and **1b-THF** have, according to SCXRD and DFT, longer Ti-THF bonds than **1a**, which indicate a weakly bonded THF ligand and a higher rate of formation of the active species and/or, by analogy to other catalytic systems [46,47,48,49,50,52,81], a faster dissociation of the growing polymer chains during the copolymerization. The length of the Ti-THF bond, in turn, does not correlate in a straightforward fashion with the net electron donation from the NHC moiety to the metal center (which are very similar; see Table 2) or with the length or strength of the Ti-NHC bond. The latter bond appears to influence the lability of the THF ligand more indirectly, via the Ti-O*i*Pr bond *trans* to the THF. For example, the long Ti-C_carbene_ bond in **1b-THF** results in a short Ti-O*i*Pr bond and, due to *trans* influence, a long and presumably weak Ti-THF bond.

## 3. Materials and Methods 

### 3.1. Experimental Details 

All operations were performed with rigorous exclusion of moisture and air, using standard Schlenk-line system and glovebox techniques under argon atmosphere (MB Braun MB200B-G, <1 ppm O_2_ and <1 ppm H_2_O). Hexane, toluene, THF and dichloromethane were purified, by using Grubbs columns (MBraun solvent purification system). Pentane, C_6_D_6_, CDCl_3_, and CHO were degassed and dried overnight over sodium or CaH_2_, back transferred and then employing the freeze-pump-thaw procedure. All chemicals were obtained from Sigma-Aldrich/Merck and used as received. Proligands **a**, **b**, and **c** [26,67,68,82,83], compounds ([*κ*^3^*-*O,C,O]-^Is^NHC)TiCl(O*i*Pr)(THF) **1c** [38] and ([*κ*^3^*-*O,C,O]-^Is^NHC)HfCl(O*i*Pr)(THF) **3c** [25,45] were prepared according to the literature procedures. [PPN]Cl was recrystallized prior to use [84]. Carbon dioxide purity grade (99.999%) was purified through copper oxide on alumina and molecular sieves (3 Å).

The NMR spectra (Bruker, Billerica, MA, USA) of air and moisture sensitive compounds were recorded by using J. Young valve NMR tubes at 25 °C on a Bruker-BIOSPIN-AV500 ultrashield 500 plus (5 mm BBO with z-gradient BTO, ^1^H: 500.13 MHz; ^13^C: 125.75 MHz), and a Bruker Ascend AV850 III HD (5 mm triple resonance CryoProbe, ^1^H: 850.13 MHz; ^13^C: 213.77 MHz). ^1^H and ^13^C shifts are referenced to internal solvent resonances and reported in parts per million relative to TMS. DRIFT spectra (Thermo Nicolet, Madison, WI, USA) were recorded by using a Nicolet protégé 460 ESP FTIR spectrometer and a DRIFT cell (KBr window) under argon atmosphere. The spectra were averaged over 64 scans; the resolution was ± 4 cm^−1^. Elemental analysis of C, H and N elements was performed on an Elementar Vario EL III. GPC-SEC (Viscotek-Malvern, Worcestershire, UK) was measured, to determine *M*_n_ and *M*_w_ of the PCHC polymers obtained from the catalytic testing, from Viscotek. Narrow polystyrene PS-99K (*M*_w_ = 99.284 kg mol^−1^, *M*_n_ = 97.381 kg mol^−1^ and IV = 0.477 dL g^−1^) and all calibration standards were obtained from Malvern PolyCAL. Approx. 30 mg of each polymer, obtained from the catalytic testing, were dissolved in exactly 10 mL THF (containing 250 ppm BHT inhibitor). The sample solutions (≈ 3.0 mg mL^−1^ in THF) were filtered through syringe filter Whatmann (0.45 µm pore size) prior to injection. Chromatographic separation was performed at a column temperature of 30 °C with a flow rate of 1 mL min^−1^. SEC was performed with a pump supplied by Viscotek (GPCmax), employing two ViscoGel columns. Signals were detected by means of a triple detection array (TDA 302) and calibrated against polystyrene standards (*Ɖ* < 1.2, from 0.12–940 kg mol^−1^). 

### 3.2. Synthesis of ([κ^3^-O,C,O]-^I^NHC)TiCl(OiPr)(THF) ***1a***

In a glovebox, to a stirred solution of Ti(O*i*Pr)_4_ (28 mg, 0.098 mmol) in 7 mL THF precooled at −30 °C was added dropwise a solution of one equiv. of **a** (50 mg, 0.098 mmol) in THF (12 mL) at −30 °C. The colorless mixture immediately turned yellow-orange upon addition of Ti(O*i*Pr)_4_ and was stirred 2 h at room temperature, then dried under vacuum. The yellow solid was washed with small fraction of hexane (3 × 2 mL) and all volatiles were removed under vacuum affording yellow powder **1a**. Yield = 86%. Yellow crystals of **1a** suitable for SCXRD analysis (Bruker-AXS, Madison, WI, USA) could be obtained from THF: hexane (1:3) at −30 °C after one week. Anal. Calcd for C_38_H_57_N_2_O_4_Ti: C, 66.22; H, 8.34; N, 4.06%. Found: C, 65.69; H, 7.82; N, 4.01%. Despite several attempts, no better elemental analysis data could be obtained. ^1^H NMR (500.13 MHz, C_6_D_6_, 25 °C): *δ*, 7.60 (d, *J* = 2.2 Hz, 2H, Ar-*H*), 7.17 (d, *J* = 2.2 Hz, 2H, Ar-*H*), 6.99 (s, 2H, NC*H*), 4.76 (sept, *J* = 6.1 Hz, 1H, O-C*H*(CH_3_)_2_), 3.58 (m, 4H, THF), 1.93 (s, 18H, *t*Bu), 1.41 (m, 4H, THF), 1.35 (s, 18H, *t*Bu), 0.94 (d, *J* = 6.1 Hz, 6H, O-CH(C*H*_3_)_2_) ppm. ^13^C{^1^H} NMR (125.77 MHz, C_6_D_6_, 25 °C): *δ*, 184.0 (N*C*N), 151.8 (*C*_q_, Ar), 141.5 (*C*_q_, Ar), 139.9 (*C*_q_, Ar), 127.1 (*C*_q_, Ar), 122.7 (*C*H-Ar), 116.2 (N*C*H), 112.3 (*C*H-Ar), 84.0 (O-*C*H(CH_3_)_2_), 67.9 (THF), 36.3 (*C*_q_, *t*Bu), 34.7 (*C*_q_, *t*Bu), 31.8 (*C*H, *t*Bu), 30.4 (*C*H, *t*Bu), 26.0 (O-CH(*C*H_3_)_2_), 25.8 (THF) ppm. DRIFT (KBr, ν/cm^−1^, [4000–400]): 2958vs, 2930s, 2906s, 2867m, 1477s, 1447s, 1390vw, 1361w, 1322s, 1270m, 1253w, 1115s, 1105m, 1009m, 923vw, 861s, 844w, 773w, 764vw, 571m, 454w.

### 3.3. Synthesis of ([κ^3^-O,C,O]-^Bz^NHC)TiCl(OiPr) ***1b***

In a glovebox, to a stirred solution of Ti(O*i*Pr)_4_ (50.5 mg, 0.178 mmol) in 5 mL THF precooled at −30 °C was added dropwise a solution of one equiv. of **b** (100 mg) in THF (12 mL) at −30 °C. The colorless mixture immediately turned yellow upon addition of Ti(O*i*Pr)_4_ and was stirred 2 h at room temperature, then dried under vacuum. The yellow solid was washed with small fraction of hexane (3 × 2 mL) and all volatiles were removed under vacuum affording yellow powder **1b**. Yield = 84%. Yellow crystals of THF adduct of **1b** (**1b-THF**) suitable for SCXRD analysis could be obtained from a mixture of THF:pentane (1:3) at −30 °C after 2 days. Anal. Calcd for C_42_H_59_ClN_2_O_4_Ti: C, 68.24; H, 8.04; N, 3.79%. Found: 68.36; H, 8.08; N, 4.15%. ^1^H NMR (500.13 MHz, C_6_D_6_, 25 °C): *δ*, 7.90 (m, 2H, Ar_bz_-*H*), 7.73 (d, *J* = 2.3 Hz, 2H, Ar-*H*), 7.62 (d, *J* = 2.3 Hz, 2H, Ar-*H*), 7.00 (m, 2H, Ar_bz_-*H*), 4.35 (sept, *J* = 6.2 Hz, 1H, O-C*H*(CH_3_)_2_), 1.93 (s, 18H, *t*Bu), 1.33 (s, 18H, *t*Bu), 0.80 (d, *J* = 6.2 Hz, 6H, O-CH(C*H*_3_)_2_) ppm. ^13^C{^1^H} NMR (125.77 MHz, C_6_D_6_, 25 °C): *δ*, 195.2 (N*C*N), 152.9 (*C*_q_, Ar), 141.3 (*C*_q_, Ar), 139.6 (*C*_q_, Ar), 133.3 (*C*_q_, Ar), 126.2 (*C*_q,_ Ar), 125.3 (*C*H-Ar), 122.9 (*C*H-Ar), 116.2 (*C*H-Ar), 114.5 (*C*H-Ar), 86.4 (O-*C*H(CH_3_)_2_), 36.3 (*C*_q_, *t*Bu), 34.7 (*C*_q_, *t*Bu), 31.7 (*C*H_3_, *t*Bu), 30.3 (*C*H_3_, *t*Bu), 25.6 (O-CH(*C*H_3_)_2_) ppm. DRIFT (KBr, ν/cm^−1^, [4000–400]): 2960vs, 2928s, 2869m, 1552m, 1483s, 1469m, 1441s, 1380m, 1364m, 1309m, 1291m, 1269m, 1256 m, 1202vw, 1119s, 1019m, 918w, 857s, 803vw, 752m, 701w, 637w, 576m, 495w, 418w. 

### 3.4. Synthesis of [κ^3^-O,C,O]-(^I^NHC)Ti(OiPr)_2_ ***2a***

To a solution of **1a** (26 mg, 0.037 mmol) in 5 mL THF precooled at –30 °C was added dropwise 1.1 equiv. of LiO*i*Pr (2.7 mg, 0.041 mmol) dissolved in 3 mL THF. The solution was stirred at room temperature overnight, then dried under vacuum and extracted with hexane. The bright yellow solution mixture was centrifuged, filtered, and then dried under vacuum affording a pale-yellow powder **2a**. Yield = 78%. Orange crystal of **2a** suitable for SCXRD analysis could be obtained from hexane at −30 °C after 2 days. Anal. Calcd for C_37_H_56_N_2_O_4_Ti: C, 69.36; H, 8.81; N, 4.37%. Found: C, 68.97; H, 8.54; N, 4.00%. ^1^H NMR (500.13 MHz, C_6_D_6_, 25 °C): *δ*, 7.61 (br d, *J* = 2.3 Hz, 2H, Ar*H*), 7.18 (br d, *J* = 2.3 Hz, overlapping with benzene signal, Ar*H*), 7.01 (s, overlapping with spinning side band, NC*H*), 5.07 (sept, *J* = 6.0 Hz, 2H, O-C*H*(CH_3_)_2_), 1.92 (s, 18H, *t*Bu), 1.39 (s, 18H, *t*Bu), 1.31 (d, *J* = 6.0 Hz, 12H, O-CH(C*H*_3_)_2_) ppm. ^13^C{^1^H} NMR (213.77 MHz, C_6_D_6_, 25 °C): *δ*, 183.6 (N*C*N), 152.7 (*C*_q_, Ar), 141.5 (*C*_q_, Ar), 139.4 (*C*_q_, Ar), 127.2 (*C*_q_, Ar), 122.3 (*C*H-Ar), 116.3 (N*C*H), 112.9 (*C*H-Ar), 77.9 (O-*C*H(CH_3_)_2_), 36.3 (*C*_q_, *t*Bu), 34.6 (*C*_q_, *t*Bu), 31.9 (*C*H_3_, *t*Bu), 30.5 (*C*H_3_, *t*Bu), 26.9 (O-CH(*C*H_3_)_2_) ppm. DRIFT (KBr, ν/cm^−1^, [4000–400]): 2957vs, 2918s, 2900s, 2861m, 1480vs, 1450s, 1391vw, 1361w, 1321s, 1272m, 1254w, 1197w, 1125m, 1007m, 922vw, 850m, 799vw, 771w, 722w, 662vw, 558w, 454w.

### 3.5. Synthesis of ([κ^3^-O,C,O]-^Bz^NHC)Ti(OiPr)_2_ ***2b***

To a solution of **1b** (80 mg, 0.108 mmol) in 5 mL THF precooled at −30 °C was added dropwise 1.2 equiv. of LiO*i*Pr (8.3 mg, 0.126 mmol) dissolved in 3 mL THF. The yellow solution was stirred at room for 2 h, then dried under vacuum and extracted with hexane. The pale orange solution mixture was centrifuged, filtered, and then dried under vacuum affording a pale-yellow powder **2b**. Yellow crystals of **2b** suitable for SCXRD analysis could be obtained from hexane at –30 °C after 2 days. Yield = 94%. Anal. Calcd for C_41_H_58_N_2_O_4_Ti.1/2THF: C, 70.19; H, 8.63; N, 3.81%. Found: C, 69.64; H, 8.63; N, 3.79%. ^1^H NMR (500.13 MHz, C_6_D_6_, 25 °C): *δ*, 7.87 (m, 2H, Ar_bz_-*H*), 7.75 (d, *J* = 2.3 Hz, 2H, Ar-*H*), 7.64 (d, *J* = 2.3 Hz, 2H, Ar-*H*), 6.96 (m, 2H, Ar_bz_-*H*), 4.94 (sept, *J* = 6.1 Hz, 1H, O-C*H*(CH_3_)_2_), 1.92 (s, 18H, *t*Bu), 1.37 (s, 18H, *t*Bu), 1.27 (d, *J* = 6.1 Hz, 6H, O-CH(C*H*_3_)_2_) ppm. ^13^C{^1^H} NMR (125.77 MHz, C_6_D_6_, 25 °C): *δ*, 198.1 (N*C*N), 153.7 (*C*_q_, Ar), 139.5 (*C*_q_, Ar), 139.0 (*C*_q_, Ar), 133.6 (*C*_q_, Ar), 126.9 (*C*_q_, Ar), 124.5 (*C*H-Ar), 122.3 (*C*H-Ar), 116.8 (*C*H-Ar), 114.2 (*C*H-Ar), 78.9 (O-*C*H(CH_3_)_2_), 36.3 (*C*_q_, *t*Bu), 34.6 (*C*_q_, *t*Bu), 31.9 (*C*H, *t*Bu), 30.4 (*C*H, *t*Bu), 26.5 (O-CH(*C*H_3_)_2_) ppm. DRIFT (KBr, ν/cm^−1^, [4000–400]): 2960vs, 2928s, 2865m, 1566m, 1485s, 1465m, 1431s, 1370m, 1362m, 1309m, 1325w, 1289m, 1269m, 1256m, 1236w, 1226w, 1198vw, 1163m, 1123s, 1013s, 922vw, 878w, 853s, 797vw, 766w, 752m, 695w, 641w, 616w, 592w, 560m, 485w.

### 3.6. Synthesis of ([κ^3^-O,C,O]-^Bz^NHC)HfCl(OiPr)(THF) ***3b***

In a glovebox, Hf(O*i*Pr)_4_(HO*i*Pr) (76.4 mg, 0.160 mmol) in 7 mL THF was added dropwise over one hour to ligand **b** (80 mg, 0.142 mmol) dissolved in 20 mL THF at room temperature. The solution immediately turned pale yellow and then completely colorless after stirring for 24 h. The reaction mixture was dried under vacuum and extracted with hexane. The colorless solution mixture was centrifuged, filtered, washed with pentane, and then dried under vacuum affording a yellow-white powder corresponding to compound **3b** along with side products. Yield ≈ 86% (based on ^1^H NMR data). Colorless crystals of **3b** suitable for SCXRD analysis could be obtained from pentane at −30 °C after 3 days. This compound could never be isolated in pure form even after repeated washings with hydrocarbon solvents. Major compound **3b**: ^1^H NMR (500.13 MHz, C_6_D_6_, 25 °C): *δ*, 8.01 (m, 2H, Ar_bz_-*H*), 7.72 (d, *J* = 2.4 Hz, 2H, Ar-*H*), 7.68 (d, *J* = 2.4 Hz, 2H, Ar-*H*), 7.01 (m, 2H, Ar_bz_-*H*), 4.56 (sept, *J* = 6.1 Hz, 1H, O-C*H*(CH_3_)_2_), 3.47 (m, 4H, THF), 1.89 (s, 18H, *t*Bu), 1.36 (s, 18H, *t*Bu), 1.10 (d, *J* = 6.1 Hz, 6H, O-CH(C*H*_3_)_2_), 0.67 (br s, 4H, THF) ppm. ^13^C{^1^H} NMR (213.77 MHz, C_6_D_6_, 25 °C): *δ*, 201.8 (N*C*N), 152.2 (*C*_q_, Ar), 141.1 (*C*_q_, Ar), 139.3 (*C*_q_, Ar), 134.2 (*C*_q_, Ar), 126.9 (*C*_q_, Ar), 124.7 (*C*H-Ar), 123.2 (*C*H-Ar), 117.9 (*C*H-Ar), 114.0 (*C*H-Ar), 73.7 (THF), 70.7 (O-*C*H(CH_3_)_2_), 36.2 (*C*_q_, *t*Bu), 34.6 (*C*_q_, *t*Bu), 31.8 (*C*H_3_, *t*Bu), 30.4 (*C*H_3_, *t*Bu), 27.0 (O-CH(*C*H_3_)_2_), 25.0 (THF) ppm.

### 3.7. Synthesis of ([κ^3^-O,C,O]-^Bz^NHC)Hf(OiPr)_2_(THF) ***4b***

In a glovebox, to a solution of **3b** (37.2 mg, 0.043 mmol) in 10 mL THF was added dropwise 1.1 equiv. LiO*i*Pr (3.1 mg, 0.047 mmol) dissolved in 5 mL THF. The solution immediately turned yellow and then completely colorless after stirring at room temperature for 24 h. The reaction mixture was dried under vacuum and extracted with hexane. The colorless solution mixture was centrifuged, filtered, washed with pentane, and then dried under vacuum affording a white powder corresponding to compound **4b** as major compound in quantitative yield along with minor unidentified side compounds. This compound could never be isolated in pure form even after repeated washings with hydrocarbon solvents. Major compound **4b**: ^1^H-NMR (500.13 MHz, C_6_D_6_, 25 °C): *δ*, 7.98 (m, 2H, Ar_bz_-*H*), 7.72 (d, *J* = 2.7 Hz, 2H, Ar-*H*), 7.68 (d, *J* = 2.7 Hz, 2H, Ar-*H*), 7.01 (m, 2H, Ar_bz_-*H*), 4.63 (br m, 2H, O-C*H*(CH_3_)_2_), 3.42 (br m, 4H, THF), 1.90 (s, 18H, *t*Bu), 1.37 (s, 18H, *t*Bu), 1.31 (6H, O-CH(C*H*_3_)_2_), 1.07 (br m, 4H, THF) ppm. ^13^C{^1^H} NMR (213.77 MHz, C_6_D_6_, 25 °C): *δ*, 206.2 (N*C*N), 153.1 (*C*_q_, Ar), 140.7 (*C*_q_, Ar), 138.7 (*C*_q_, Ar), 134.5 (*C*_q_, Ar_bz_), 127.1 (*C*_q_, Ar), 124.2 (*C*H-Ar_bz_), 122.7 (*C*H-Ar), 118.2 (*C*H-Ar), 113.8 (*C*H-Ar_bz_), 71.5 (O-*C*H(CH_3_)_2_), 69.1 (THF), 36.3 (*C*_q_, *t*Bu), 34.5 (*C*_q_, *t*Bu), 31.9 (*C*H_3_, *t*Bu), 30.4 (*C*H_3_, *t*Bu), 28.0 (O-CH(*C*H_3_)_2_), 25.2 (THF) ppm.

### 3.8. Copolymerization of CHO and CO_2_

A detailed copolymerization procedure is described as a typical example (Table 5, Entry 1). In a glovebox, an reaction tube for low-pressure reactions equipped with a magnetic stirring bar, a solution of the [PPN]Cl cocatalyst (8 µmol) in dichloromethane (ca. 1 mL) was added under vigorous stirring to a solution of complex **1a** (8 µmol) in dichloromethane (ca. 1 mL). The mixture was stirred at ambient temperature for 15 min and the solvent was removed under vacuum (30 min). The resulting solid was then dissolved in a precooled (−30 °C) solution of CHO (10 mmol). Then to the reaction tube was added 2 bar of CO_2_ and the reaction mixture was heated to 65 °C. After 24 h, the reaction was cooled down and the pressure was released. An aliquot of the solution was taken for characterization of crude material by ^1^H-NMR spectroscopy in CDCl_3_. Then, the reaction mixture was quenched with 1 mL of acidic methanol, precipitated with methanol, and dried for several hours at 80 °C until constant weight. The yield was determined gravimetrically. 

### 3.9. X-ray Crystallographic Details

Suitable crystals for diffraction experiments were selected in a glovebox and mounted in a minimum of Parabar 10,312 oil (Hampton Research) in a nylon loop and then mounted under a nitrogen cold stream from an Oxford Cryosystems 700 series open-flow cryostat. Data collection was done on a Bruker AXS TXS rotating anode system with an APEXII Pt^135^ CCD detector (Bruker-AXS, Madison, WI, USA) using graphite-monochromated Mo Kα radiation (λ = 0.710 73 Å). Data collection and data processing were done using APEX2 [85], SAINT [86], and SADABS [87] version 2012/1, whereas structure solution and final model refinement were done using SHELXS [88] version 2013/1 or SHELXT [89] version 2014/4 and SHELXL [90] version 2014/7. Details of the crystallographic analyses for **1a**, **2a**, **1b-THF**, **1b’**, **2b**, and **3b** are given in Supplementary Tables S1 and S5 and in CIF files (CCDC reference codes 2026401-2026406). These data can be obtained free of charge via www.ccdc.cam.ac.uk/data_request/cif, or by emailing data_request@ccdc.cam.ac.uk, or by contacting The Cambridge Crystallographic Data Centre, 12 Union Road, Cambridge CB2 1EZ, UK, fax: +44 1223 336033.

### 3.10. Computational Methods

All density functional theory (DFT) calculations were performed with the Gaussian 16 suite of programs [91].

#### 3.10.1. Geometry Optimization

Geometry optimization was performed using the Gaussian 16 implementation of the generalized-gradient approximation (GGA) functional of Perdew, Burke and Ernzerhof (PBE) [92] including Grimme’s D3 empirical dispersion term [93] with revised Becke-Johnson damping parameters [94] (overall labelled PBE-D3M(BJ) for brevity). All atoms except titanium were described by Dunning’s correlation-consistent valence triple-ζ plus polarization basis sets (termed cc-pVTZ) [95,96], as retrieved from the EMSL basis set exchange database [97,98]. Titanium was described by the Stuttgart 10-electron relativistic effective core potential (termed ECP10MDF) in conjunction with its accompanying primitive basis set (8s7p6d2f1g) contracted to [6s5p3d2f1g]. Both the effective core potential and the accompanying basis set were retrieved from the Stuttgart/Cologne group website [99]. Numerical integrations were performed using the Gaussian 16 (99,590) “ultrafine” grid (keyword int = ultrafine), a pruned grid consisting of 99 radial shells and 590 angular points per shell, except when solving the coupled-perturbed Hartree-Fock equations (part of the analytical second-derivatives calculations) for which the pruned (75,302 grid) “fine” grid was used (keyword CPHF=(Grid=Fine)). Geometries were optimized using tight convergence criteria (max. force 1.5 × 10^−5^ a.u., RMS force 1.0 × 10^−5^ a.u., max. force 6.0 × 10^−5^ a.u., RMS force 4.0 × 10^−5^ a.u.), without symmetry constraints, using convergence criteria for the self-consistent field (SCF) optimization procedure that were tightened tenfold compared to the default settings. The tightened criteria were RMS change in density matrix < 1.0 × 10^−9^ and max. change in density matrix < 1.0 × 10^−7^. All stationary points were characterized by the eigenvalues of the analytically calculated Hessian matrix and confirmed to be minima.

#### 3.10.2. Single-Point Energy Calculations

All single-point energy calculations were performed with the same PBE-D3M(BJ) functional as described above for geometry optimization. For titanium, carbon, and hydrogen atoms, the basis sets were also the same as those used in the geometry optimizations. All other atoms (N, O and Cl) were described by correlation-consistent valence triple-ζ plus polarization basis sets augmented by diffuse functions (EMSL: aug-cc-pVTZ) [95,97,98,100]. Numerical integrations were performed with the “ultrafine” grid of Gaussian 16, and the SCF density-based convergence criterion was set to 10^−5^ (RMS change in density matrix < 1.0 × 10^−5^, max. change in density matrix = 1.0 × 10^−3^).

#### 3.10.3. Natural Bond Orbital Calculations

All natural bond orbital calculations were performed with the NBO7 program [74], using the wavefunction produced by the Gaussian 16 single-point energy calculation as input. Keywords used in the NBO7 job include “bndidx”, which requests the print-out of the NAO-Wiberg Bond Index array, “NBO” which requests the calculation and printing of NBO’s, and “DMNAO” which requests the natural atomic orbital density matrix. To get a comparable set of orbitals among the complexes, the Lewis structures were explicitly restricted to those shown in Supplementary Scheme S2 via the $CHOOSE input section. 

## 4. Conclusions

A series of titanium and hafnium complexes bearing unsaturated and benzannulated tridentate, bis-phenolate NHC ligands have been synthesized and characterized. The Ti-C_carbene_ distances with which these ligands bind to the metal vary considerably (by 9 pm), and these differences manifest themselves, via *trans* influence and other “ripple effects”, in significant variations also in the other metal-ligand bond distances. These structural differences and their relation to the metal-NHC bonds and the electronic properties of the ligands have been studied for titanium complexes **1a**, **1b-THF**, and **1c** using DFT and NBO analyses. The shorter Ti-C_carbene_ distance in **1a** than in the other two complexes seems to originate from stronger ligand-to-metal π-donation, whereas the corresponding σ-donation is weaker than in **1c**. In contrast, back-donation directly from the metal to the NHC ligand seems to be unimportant in these d^0^ complexes. Still, the C-N π* NHC orbitals are involved in bonding as they interact with THF and isopropoxide oxygen lone-pair donor orbitals, an interaction that appears to contribute to the tilting of the NHC ligand toward the THF in **1b-THF**.

The new complexes catalyze the copolymerization of CHO with CO_2_ under mild reaction conditions (*P*_CO2_ = 2 bar and 65 °C) to exclusively give poly(cyclohexene carbonate) product, albeit with low-to-moderate yields. Among the new complexes, the benzannulated-NHC-coordinated titanium complex (**1b-THF**) gives the most active catalyst upon activation with [PPN]Cl. Including previously reported complexes, the order among the NHC ligands in terms of catalytic activity is as follows: imidazolidin-2-ylidene (saturated) > benzimidazolin-2-ylidene (benzannulated) > imidazolin-2-ylidene (unsaturated). Although further mechanistic studies are needed to uncover the factors governing this order, it might be influenced by the inherent stability of the complexes and possibly also the lability of the THF ligand, as suggested by the variation in Ti-THF distance among the complexes. 

## Figures and Tables

**Table 1:** Interatomic distances, angles, and torsion angles from SCXRD data and from DFT-optimized geometries.

	(^I^NHC)TiCl(O*i*Pr) (THF) (1a)	(^Bz^NHC)TiCl(O*i*Pr)(THF) (1b-THF) ^a^	(^Is^NHC)TiCl (O*i*Pr)(THF) (1c) ^b^
**Bond Length (Å)** ^c^			
Ti-C_carbene_	2.1310(13)/2.144	2.221(2)/2.214	2.166(3)/2.180
Ti-Cl	2.3739(4)/2.337	2.3459(8)/2.325	2.383(1)/2.336
Ti-O*i*Pr	1.7671(11)/1.785	1.758(2)/1.789	1.779(2)/1.774
Ti-THF	2.2573(11)/2.318	2.2865(2)/2.347	2.272(2)/2.351
**Angle (°)** ^c^			
∠O_Ar_-Ti-O_Ar_	159.14(4)/157.93	159.18(8)/158.70	159.19(9)/158.23
∠Ti-O-C*_i_*_Pr_	139.95(9)/143.18	160.5(3)/140.92	154.7(3)/160.48
∠N-C_carbene_-N	105.22(11)/105.40	106.0(2)/106.20	108.8(2)/108.91
**Torsion Angle (°)** ^c^			
∠O_Ar_-C_Ar_-N-C_carbene_	5.86/8.02	27.04/22.78	3.35/4.61
∠O_Ar_-C_Ar_-N-C_carbene_	6.89/9.71	−25.40/−25.67	3.51/5.54

^a^ Selected bond distances (Å), angles, and torsion angles (°) for **1b-THF-isomer A** and for **1b-THF-isomer B**: Ti-C_carbene_ = 2.212(2), Ti-Cl = 2.3422(8), Ti-O*i*Pr = 1.7685(18), Ti-THF = 2.2867(19),: ∠O_Ar_-Ti-O_Ar_ = 155.38(8), ∠Ti-O-C*_i_*_Pr_ = 165.57(19), ∠N-C_carbene_-N = 106.5(2), ∠O_Ar_-C_Ar_-N-C_carbene_ = 24.50, ∠O_Ar_-C_Ar_-N-C_carbene_ = −26.06. ^b^ Ref. [38]. ^c^ SCXRD structure/DFT-optimized structure.

**Table 2:** NHC binding energies, natural charges, and net electron donation to Ti.

Complex	Ti-NHC Snapping Energy ^a^ (kcal mol^−1^)	NHC Fragment Charge ^b^ (e^−^)	NHC→Ti Net Donation ^c^ (№ of e^−^)
**1a**	597.3	−0.76	0.57
**1b-THF**	587.3	−0.78	0.58
**1c**	592.7	−0.76	0.58

^a^ The Ti-NHC bond energies, or bond snapping energies, were calculated from the DFT total energies (i.e., not the free energies; see Table S2) by dissociating the tridentate ligands heterolytically to frozen-geometry fragments **M1a–c** and **Ma–c**. ^b^ The NHC fragment charge is the sum of all the natural atomic charges of the tridentate NHC ligand. ^c^ The NHC**→**Ti net donation is estimated as the number of electrons needed to reach neutrality for a NHC fragment in which the atomic charges of the two oxygen atoms have been subtracted (Table S3).

**Table 3:**
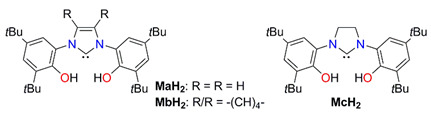
Absolute energies and electron populations of ligand natural orbitals important for the Ti-NHC interaction.

Orbital ^a^			MaH_2_^b^	MbH_2_	McH_2_
C *σ*	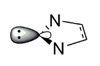	Population (№ of electrons)	1.71	1.74	1.69
		Energy (kcal mol^−1^)	−136.2	−142.0	−133.3
C-N *π*	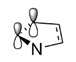	Population (№ of electrons)	1.85	1.86	1.90
		Energy (kcal mol^−1^)	−200.4	−200.3	−198.3
C-N π*	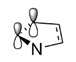	Population (№ of electrons)	0.49	0.45	0.42
		Energy (kcal mol^−1^)	−37.4	−37.4	−33.9

^a^ The backbone of the NHC shown in the orbital figures is unsaturated for **MaH_2_**, benzannulated for **MbH_2_** and saturated for **McH_2,_** see Scheme S1. ^b^ **MaH_2_**, **MbH_2_** and **McH_2_** are the neutral, model, OH-containing free-carbene ligands of the complexes **1a**, **1b-THF** and **1c**.

**Table 4:** Second-order perturbative estimates of donor-acceptor interactions in the NBO basis of **1a**, **1b-THF** and **1c**.

Complex	Donor Orbital ^a^	Acceptor Orbital	E2 (kcal mol^−1^)
	C-N π	Ti *d*_π_	2.95
**1a**	O*_i_*_Pr_ LP	C-N π*	1.01
	O_THF_ LP	C-N π*	1.26
	C-N π	Ti *d*_π_	2.44
**1b-THF**	O*_i_*_Pr_ LP	C-N π*	0.30
	O_THF_ LP	C-N π*	3.28
**1c**	C-N π	Ti *d*_π_	2.75
	C-N π	C-N π*	0.83
	O*_i_*_Pr_ LP	C-N π*	0.39
	O_THF_ LP	C-N π*	1.42

^a^ LP refers to lone pair.

**Table 5:**
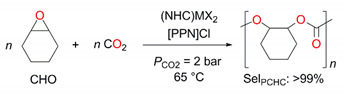
Copolymerization of CHO-CO_2_ catalyzed by titanium and hafnium NHC complexes.

Entry	Precursor ^a,b^	Yield (%) ^c^	Productivity (g_PCHC_ mol_M_^−1^ h^−1^)	TOF (h^−1^) ^d^	*M*_n_ (kg mol^−1^) ^e^	*Ɖ* ^e^
1	**1a**	37	2745	22	1.8	1.59
2	**2a**	26	1940	15	- ^g^	- ^g^
3	**1b**	49	3618	28	4.5	1.43
4	**1b** ^f^	19	6709	62	1.2	1.54
5	**2b**	48	3557	24	4.0	1.44
6	**1c** ^f^	52	18,572	116	5.9	1.41
7	**3b**	10	770	6	- ^g^	- ^g^
8	**4b**	12	897	7	- ^g^	- ^g^
9	**3c** ^f^	49	17,426	116	9.0	1.18

^a^ Catalyst preformation: addition of 1 equiv. of [PPN]Cl to the precursor in CH_2_Cl_2_ at 30 °C for 15 min and dried 30 min under vacuum. ^b^ Copolymerization conditions: 0.08 mol%_M_, 10 mmol of CHO, *P*_CO2_ = 2 bar at 65 °C for 24 h. ^c^ Determined by gravimetry. ^d^ Turnover frequency. ^e^ Determined by GPC-SEC in THF at 30 °C against polystyrene standards. ^f^ 5 h. ^g^ Not determined. Note: For all runs, the carbonate linkages are ≥99% and the selectivity in PCHC are >99% without by-products and determined by ^1^H-NMR spectroscopy in CDCl_3_.

## Supplementary Materials

The following are available online at, Figure S1: ^1^H-NMR spectrum of complex **1a**; Figure S2: ^13^C NMR spectrum of complex **1a**; Figure S3: ^1^H NMR spectrum of complex **1b**; Figure S4: ^13^C NMR spectrum of complex **1b**; Figure S5: ^1^H NMR spectrum of **1b**, **1b’** and **1b’’**; Figure S6: Molecular structure of zwitterionic compound **1b’**; Table S1: Crystal structure and refinement data for **1a**, **1b-THF** and **1b’**; Scheme S1: Complexes and ligands that have been subjected to DFT calculations; Table S2: DFT energies at the single-point level of theory; Table S3: Natural atomic charges of complexes **1a**, **1b-THF** and **1c**; Table S4: Second-order perturbative estimates of donor-acceptor interactions in the NBO basis of **1a**, **1b-THF** and **1c**; Figure S7: ^1^H NMR spectrum of complex **2a**; Figure S8: ^13^C NMR spectrum of complex **2a**; Figure S9: ^1^H NMR spectrum of complex **2b**; Figure S10: ^13^C NMR spectrum of complex **2b**; Table S5: Crystal structure and refinement data for **2a**, **2b** and **3b**; Figure S11: ^1^H NMR spectrum of complex **3b**, **3b’** and **3b’’**; Figure S12: ^1^H NMR spectrum of complex **4b**; Table S6: Interatomic distances, angles and torsion angles for 5-coordinate complexes **2a**, **2b** and **2c**; Table S7: Interatomic distances, angles and torsion angles for 6-coordinate complexes **3b** and **3c**; Scheme S2: Lewis structures used in the NBO analyses; and list of Cartesian coordinates. The following are available online, Supporting Information (pdf) containing crystal data. CIF files containing crystal data for complexes **1a**, **2a**, **1b-THF**, **1b’**, **2b**, and **3b**.
