# Supplementary material for: Unsaturated and Benzannulated N-Heterocyclic Carbene Complexes of Titanium and Hafnium: Impact on Catalysts Structure and Performance in Copolymerization of Cyclohexene Oxide with CO2
Source: Molecules. 2020 Sep 23;25(19):4364. doi: 10.3390/molecules25194364 (PMC7582562; doi:10.3390/molecules25194364)
Supplement: Supplementary file 1 [file molecules-25-04364-s001.pdf]

Unsaturated and Benzannulated *N*-Heterocyclic Carbene Complexes of Titanium and Hafnium: Impact on Catalysts Structure and Performance in Copolymerization of Cyclohexene Oxide with CO_2

Lakshmi Suresh [1,†], Ralte Lalrempuia [1,2,†], Jonas B. Ekeli [1,†], Francis Gillis-D'Hamers [1,†], Karl W. Törnroos [1], Vidar R. Jensen [1,*] and Erwan Le Roux [1,*]

[1] Department of Chemistry, University of Bergen, Allégaten 41, Bergen, Norway
[2] Current address: School of Chemical Sciences, Dublin City University, Dublin 9, Ireland
* Correspondence: Correspondence: Vidar.Jensen@uib.no; (V.R.J.) and Erwan.LeRoux@uib.no; (E.L.R.)

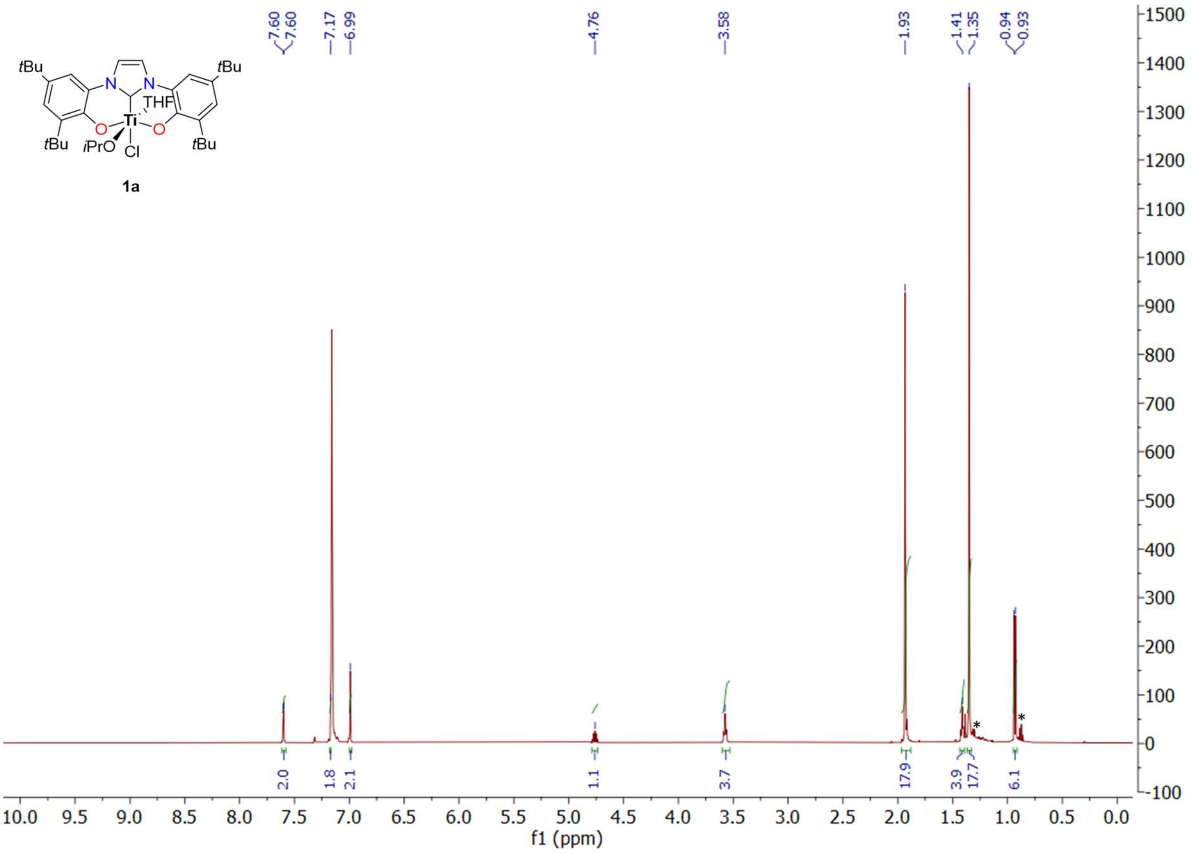

Figure S1. ^{1}H NMR spectrum of complex **1a** in C_6D_6 (residual solvent: * = pentane).

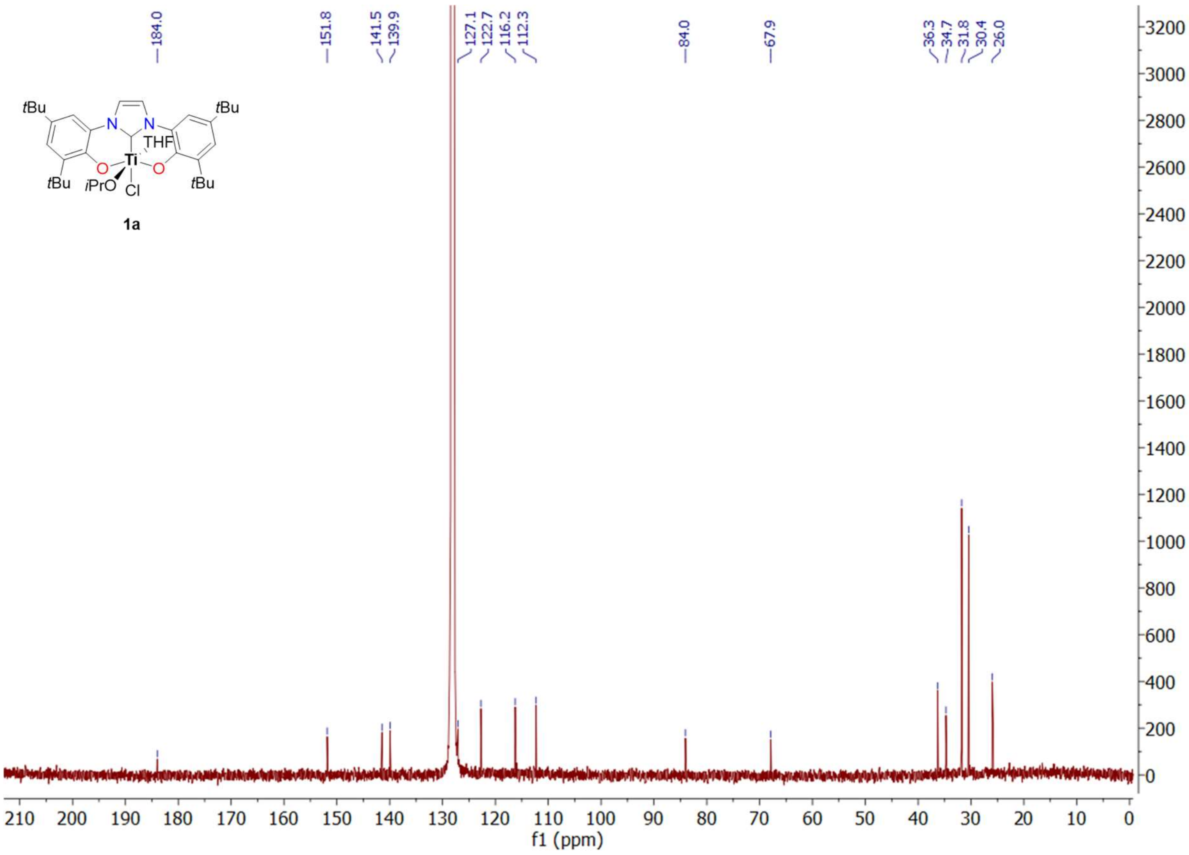

Figure S2. ^{13}C NMR spectrum of complex **1a** in C_6D_6.

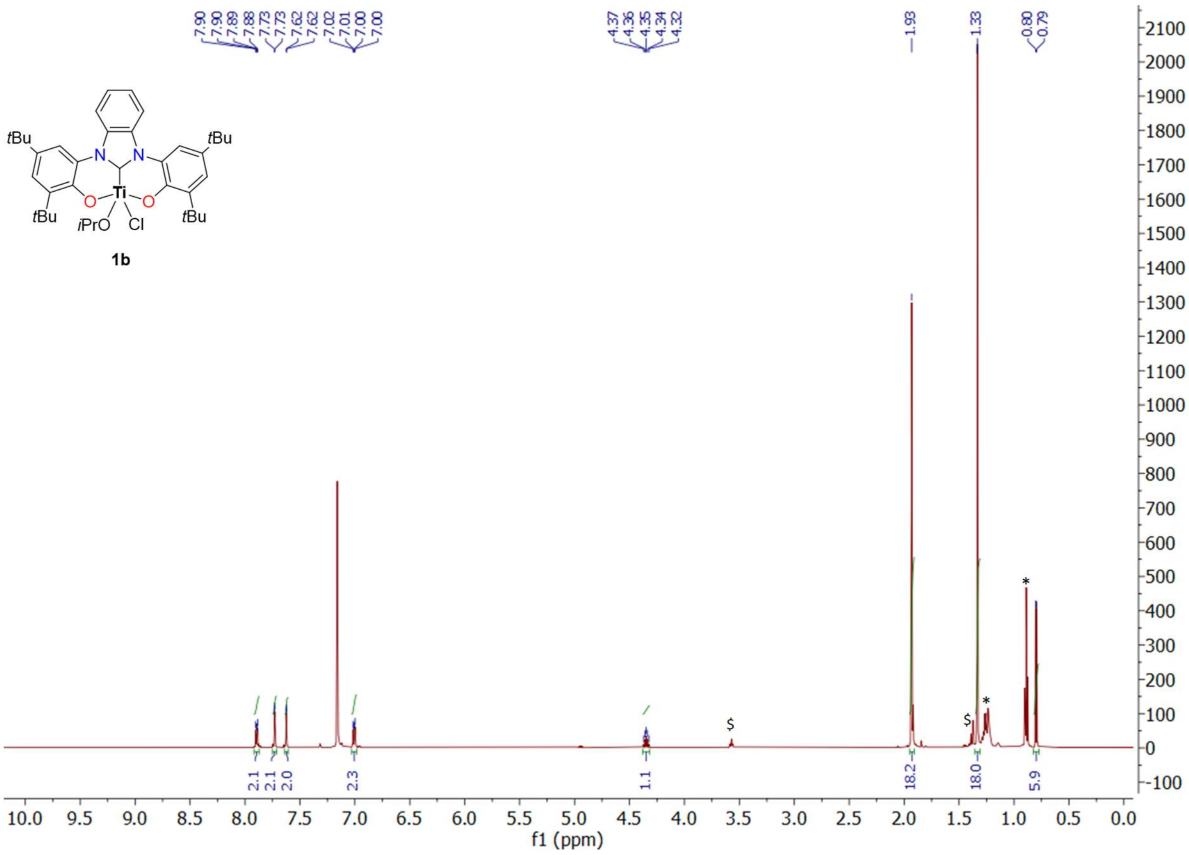

Figure S3. ^{1}H NMR spectrum of complex **1b** in C_6D_6 (residual solvents: * = hexane, $ = THF).

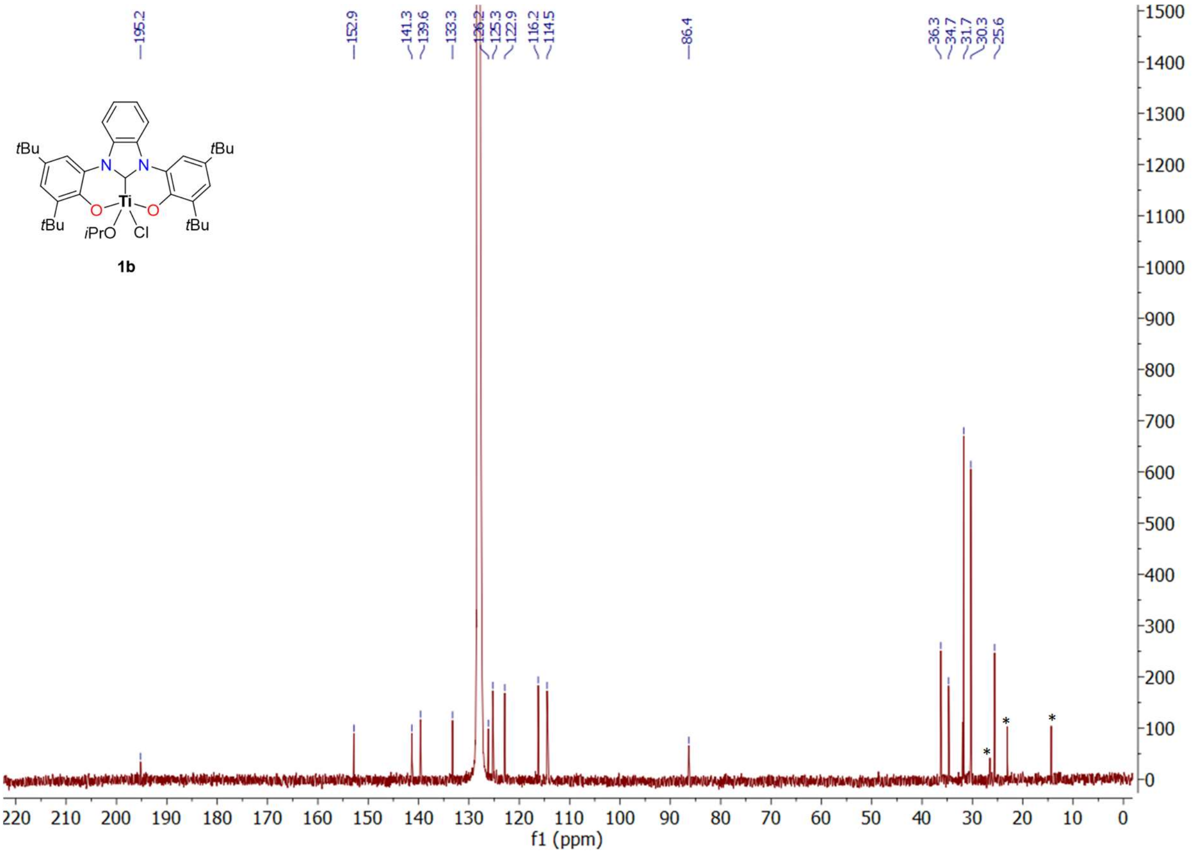

Figure S4. ^{13}C NMR spectrum of complex **1b** in C_6D_6 (residual solvent: * = hexane).

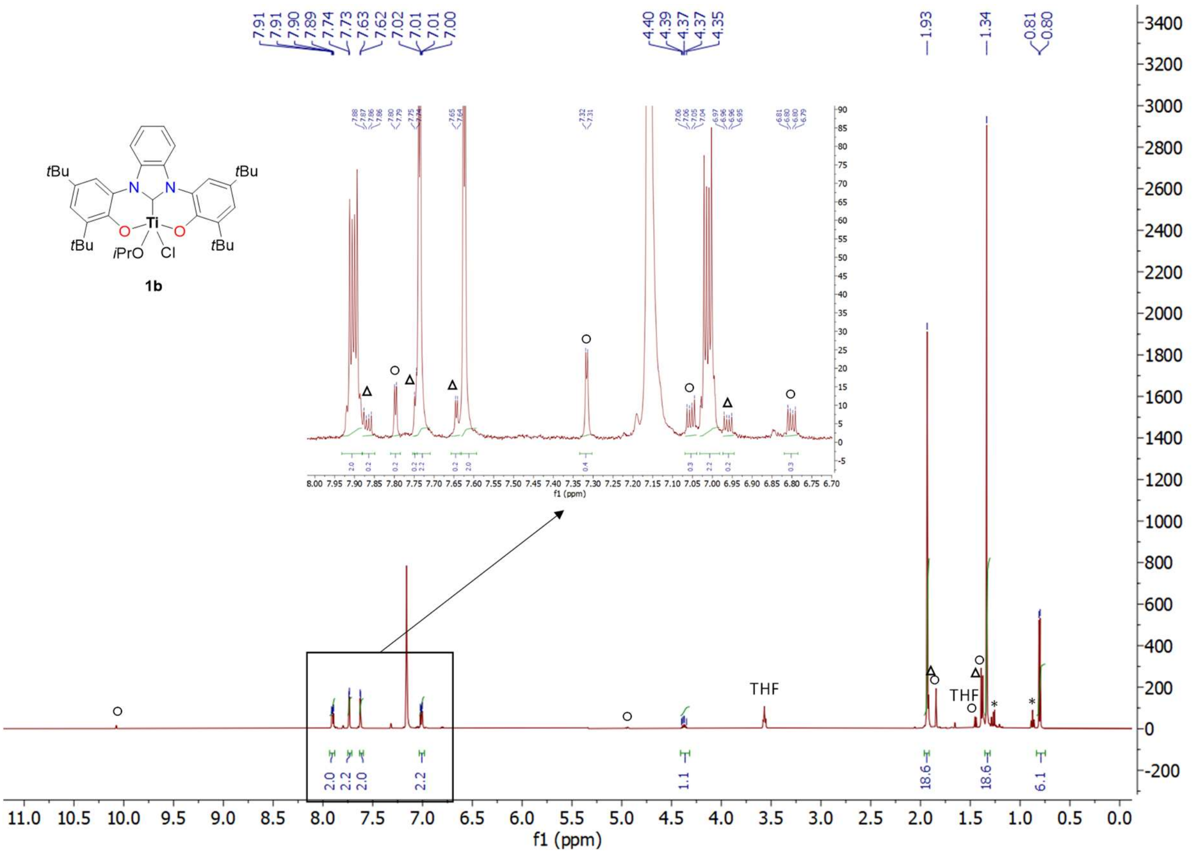

Figure S5. ^1H NMR spectrum of **1b** (major complex), **1b′** (○ = zwitterionic) and **1b′′** (Δ = homoleptic) in C_6D_6 (residual solvents: * = pentane).

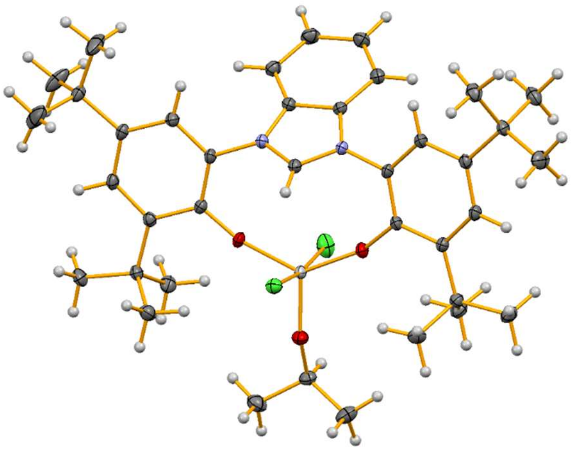

Figure S6. Molecular structure of zwitterionic compound **1b`**. A solvent toluene molecule is omitted for clarity. ADP's are given at the 50%pobability level.

Table S1. Crystal structure and refinement data for **1a**, **1b-THF** and **1b`**.

	**1a**	**1b-THF**	**1b`.C_7H_8**
Formula	$C_{38}H_{57}ClN_2O_4Ti$	$C_{42}H_{59}ClN_2O_4Ti$	$C_{45}H_{60}Cl_2N_2O_3Ti$
Formula weight	689.20	739.26	795.75
Temperature/ K	123(2)	123(2)	103(2)
Wavelength/ Å	0.71073	0.71073	0.71073
Crystal system	Monoclinic	Monoclinic	Monoclinic
Space group	$P2_1/c$ (No.14)	P21/c (No.14)	$P2_1/c$ (No.14)
a/ Å	8.2605(4)	10.5781(10)	11.3722(8)
b/ Å	32.2931(15)	49.578(5)	17.4005(12)
c/ Å	15.8217(7)	17.2457(19)	21.7582(15)
α/ Å	90	90	90
β/ Å	98.2506(7)	107.5630(10)	97.3740(2)
γ/ Å	90	90	90
V/ $Å^3$	4176.9(3)	8622.8(15)	4269.9(5)
Z	4	8	4
d / g cm^{-3}	1.096	1.139	1.238
F(000)	1480	3168	1696
Absorption coefficient/ mm^{-1}	0.305	0.299	0.366
θ Range for data collection/ °	1.812 to 30.031	1.747 to 26.385	1.888 to 30.043
Reflections collected	70019	111295	69973
Independent reflections	12232 [R_{int} = 0.0439]	17641 [R_{int} = 0.0710]	12470 [R_{int} = 0.0756]
Completeness to θmax	100%	100%	100%
Data/ restraints/ parameters	12232/7/438	17641/159/1055	12470/0/496
Goodness-of-fit on F^2	1.075	1.074	1.025
Final R indices [I > 2σ(I)]	R_1 = 0.0500, wR_2 = 0.1324	R_1 = 0.0580, wR_2 = 0.1382	R_1 = 0.0501, wR_2 = 0.01331
R indices (all data)	R_1 = 0.0564, wR_2 = 0.1375	R_1 = 0.0751, wR_2 = 0.1462	R_1 = 0.0702 wR_2 = 0.1488
Largest diff. peak/ hole/ e $Å^3$	1.044 and -0.485	0.719 and -0.653	1.224 and -1.601

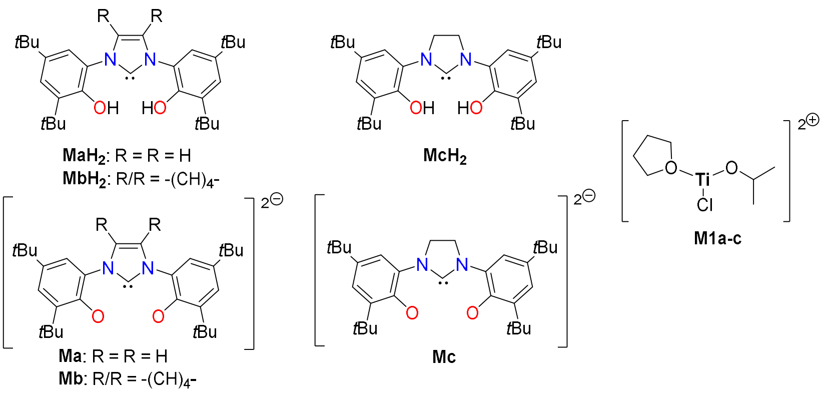

Scheme S1. Complexes and ligands that have been subjected to DFT calculations, with "**M**" indicating the model, or computational, nature of the compounds. **MaH$_2$**, **MbH$_2$** and **McH$_2$** are the neutral, model, OH-containing free-carbene ligands of the complexes **1a**, **1b-THF** and **1c**. **M1a-c** are the [TiCl(O*i*Pr)(THF)]$^{2+}$ fragments of resulting from heterolytic dissociation of the corresponding dianionic ligands **Ma**, **Mb** and **Mc** from complexes **1a**, **1b-THF** and **1c**. The geometries of **M1a-c**, **Ma**, **Mb** and **Mc** are those of the corresponding complexes.

Table S2. DFT energies at the single-point level of theory.

Structure	DFT energy (a.u.)	Structure	DFT energy (a.u.)
**1a**	-2409.80199313	**MaH$_2$**	-1466.46943745
**1b-THF**	-2563.31555107	**MbH$_2$**	-1619.98071071
**1c**	-2410.9990223	**McH$_2$**	-1467.66741041
**Ma**	-1465.19760679	**M1a**	-943.652459677
**Mb**	-1618.73449683	**M1b**	-943.645078628
**Mc**	-1466.39782849	**M1c**	-943.656685534

Table S3. Natural atomic charges of complexes **1a**, **1b-THF** and **1c**.

	Atomic Charges (e$^-$)		
**Atom**	**1a**	**1b-THF**	**1c**
Ti	1.37	1.41	1.36
$C_{carbene}$	0.17	0.21	0.24
$O_{i\text{-Pr}}$	-0.63	-0.65	-0.63
O_{THF}	-0.57	-0.58	-0.57
Cl	-0.44	-0.43	-0.44
O_{NHC}	-0.68, -0.65	-0.68, -0.68	-0.68, -0.66

Table S4. Second-order perturbative estimates of donor-acceptor interactions in the NBO basis of **1a**, **1b-THF** and **1c**.

Complex	Donor orbital[a]	Acceptor orbital[b]	E2 (kcal mol^{-1})
**1a**	O_{iPr} LP	Ti *d*	215.15
	O_{THF} LP	Ti *d*	44.62
**1b-THF**	O_{iPr} LP	Ti *d*	210.85
	O_{THF} LP	Ti d	43.67
**1c**	O_{iPr} LP	Ti *d*	210.51
	O_{THF} LP	Ti *d*	43.84

[a] LP refers to lone pair. [b] Ti *d* refers to the sum of all the lone *d*-vacant orbitals of Ti.

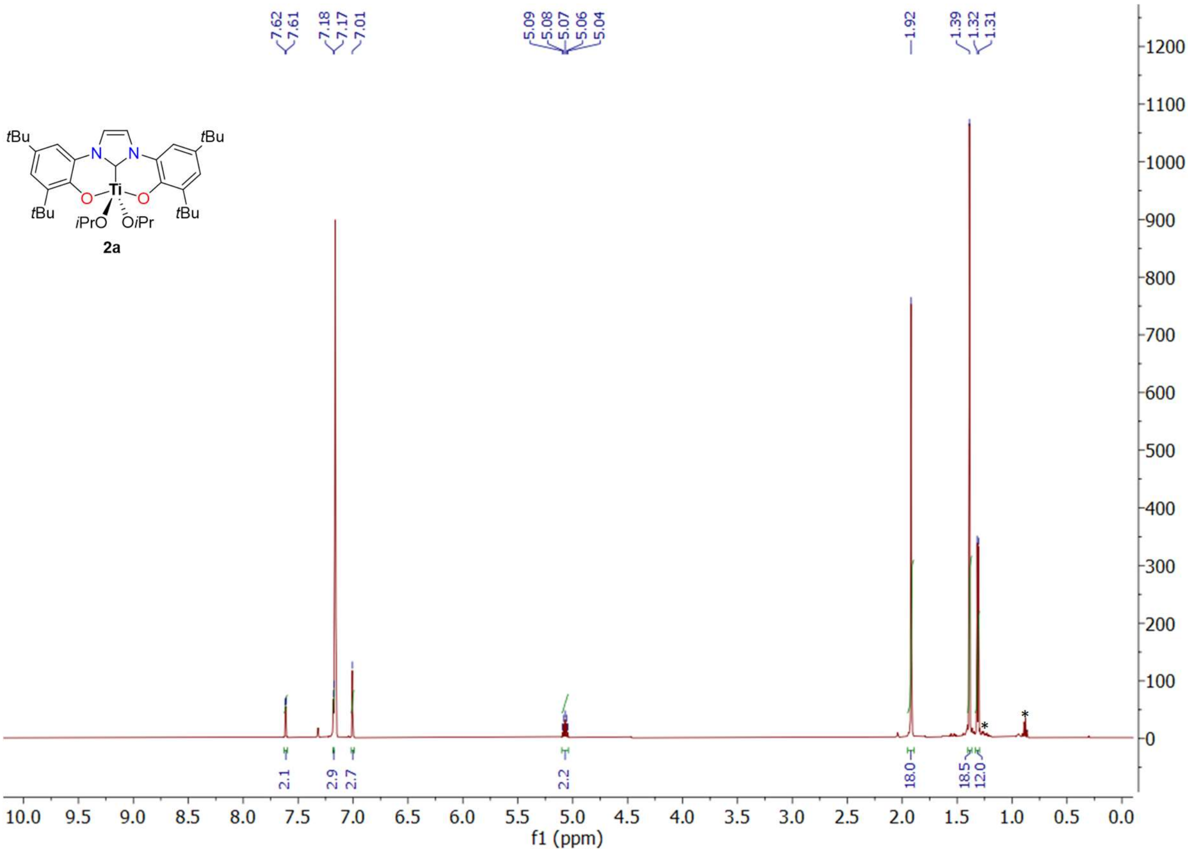

Figure S7. ^{1}H NMR spectrum of complex **2a** in C_6D_6 (residual solvents: * = pentane).

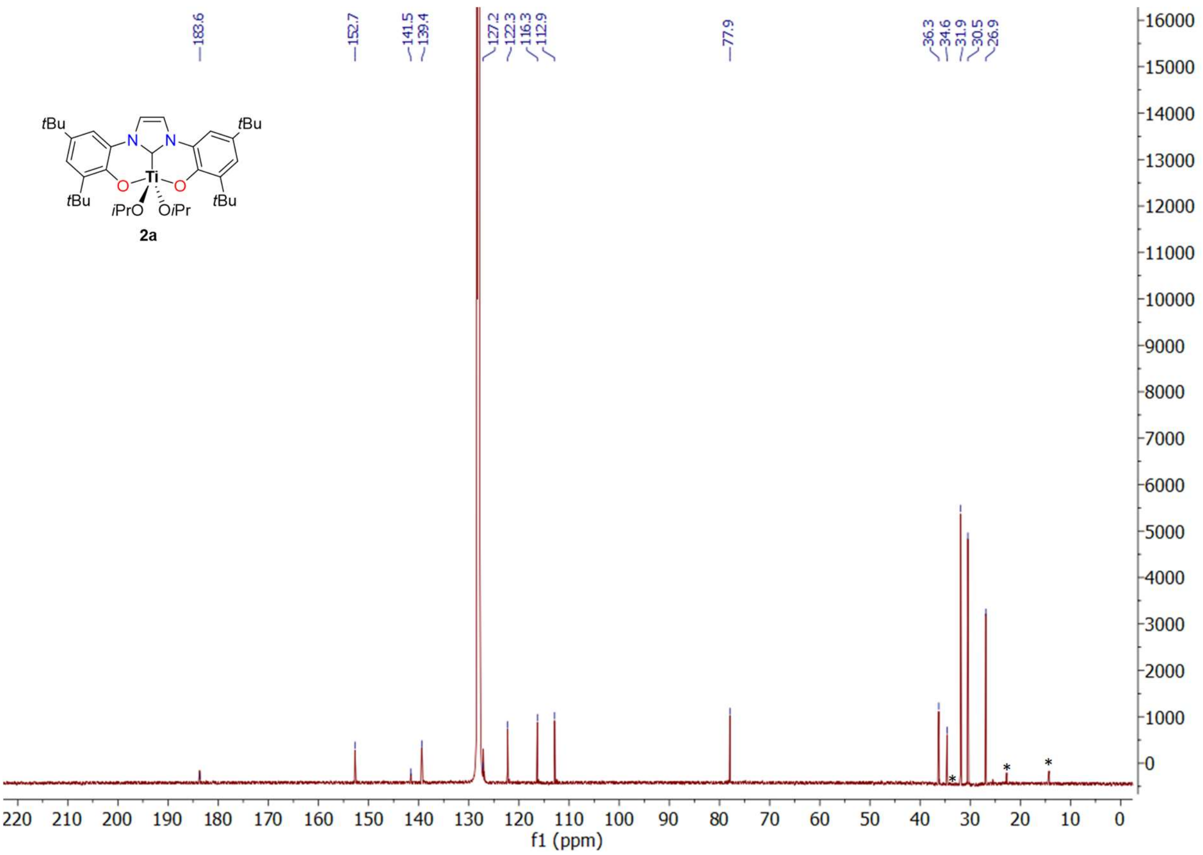

Figure S8. ^{13}C NMR spectrum of complex **2a** in C_6D_6 (residual solvents: * = pentane).

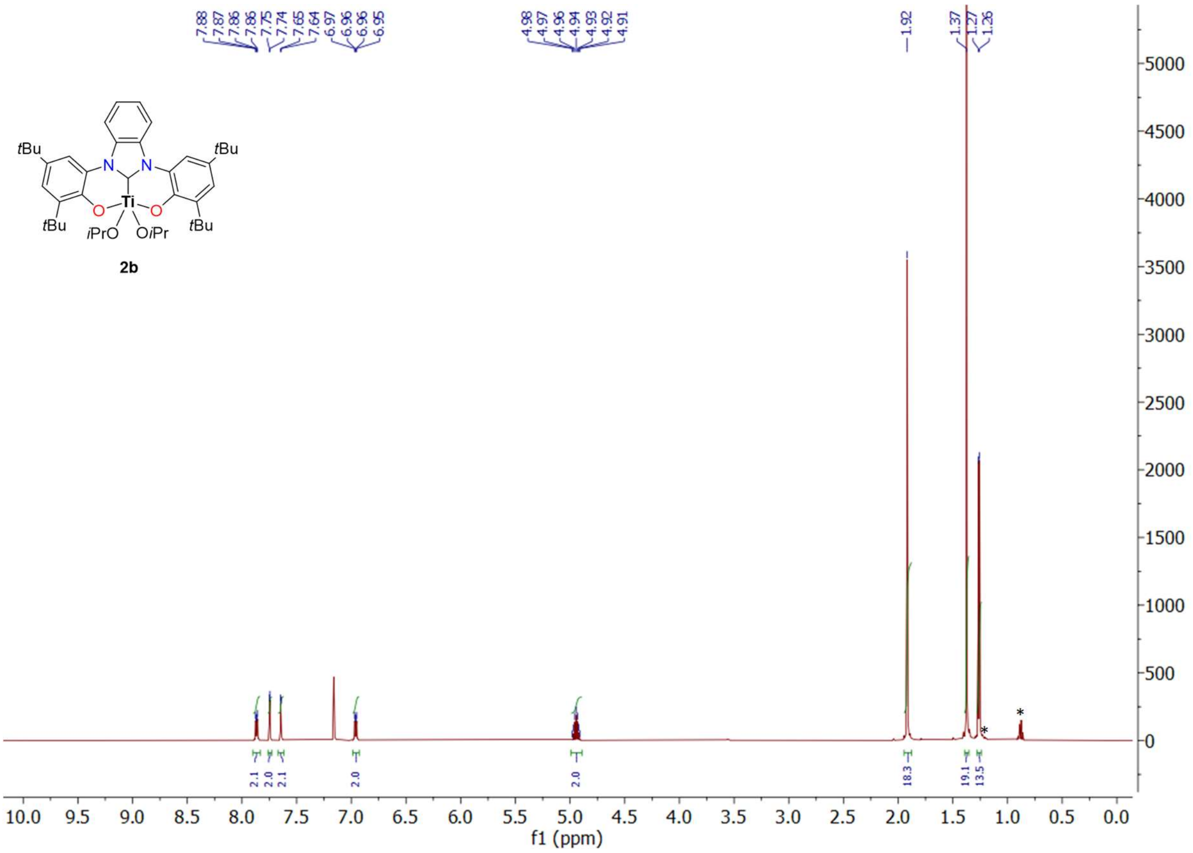

Figure S9. ^{1}H NMR spectrum of complex **2b** in C_6D_6 (residual solvents: * = pentane).

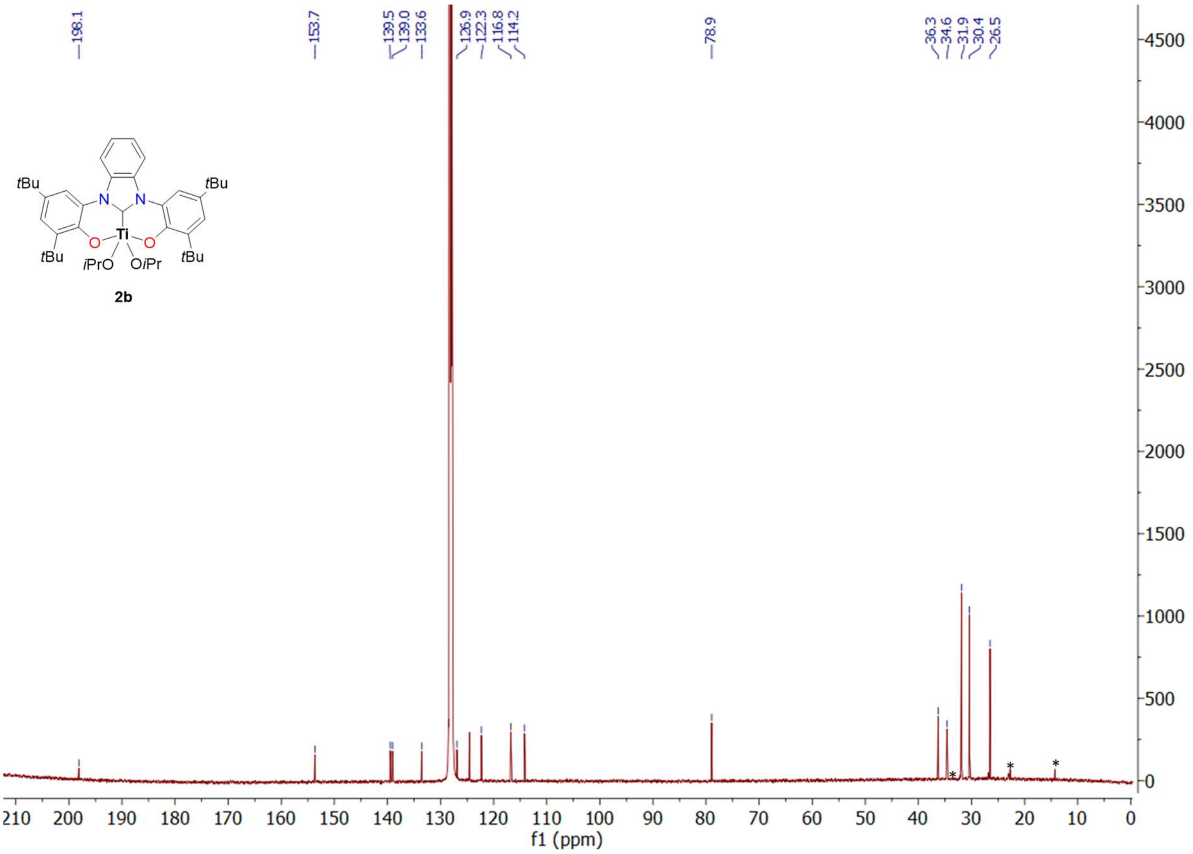

Figure S10. ^{13}C NMR spectrum of complex **2b** in C_6D_6 (residual solvents: * = pentane).

Table S5. Crystal structure and refinement data for **2a**, **2b** and **3b**.

	**2a**	**2b**	**3b.C_5H_{12}**
Formula	$C_{37}H_{56}N_2O_4Ti$	$C_{41}H_{58}N_2O_4Ti$	$C_{47}H_{71}ClHfN_2O_4$
Formula weight	640.73	690.79	941.99
Temperature/ K	103(2)	173(2)	103(2)
Wavelength/ Å	0.71073	0.71073	0.71073
Crystal system	Monoclinic	Triclinic	Monoclinic
Space group	$P2_1/n$ (No.14)	$P\bar{1}$ (No.2)	$P2_1/c$ (No.14)
a/ Å	13.499(2)	10.6656(16)	14.2090(13)
b/ Å	14.515(2)	11.3970(17)	12.1084(11)
c/ Å	19.514(3)	18.162(3)	27.547(3)
α/ Å	90	99.699(3)	90
β/ Å	109.345(2)	107.003(2)	104.3970(10)
γ/ Å	90	98.621(2)	90
V/ $Å^3$	3607.6(10)	2034.0(6)	4586.8(7)
Z	4	2	4
d / g cm^{-3}	1.180	1.128	1.364
F(000)	1384	744	1952
Absorption coefficient/ mm^{-1}	0.276	0.249	2.375
θ Range for data collection/ °	1.787 to 26.489	1.855 to 25.386	1.848 to 30.061
Reflections collected	45284	23992	75760
Independent reflections	7432 [R_{int} = 0.0609]	7461 [R_{int} = 0.0522]	13421 [R_{int} = 0.0680]
Completeness to θmax	99.7%	100%	100%
Data/ restraints/ parameters	7432/46/480	7461/71/473	13421/0/512
Goodness-of-fit on F^2	1.091	1.033	1.064
Final R indices [I > 2σ(I)]	R_1 = 0.0641, wR_2 = 0.1799	R_1 = 0.0566, wR_2 = 0.1532	R_1 = 0.0410, wR_2 = 0.0987
R indices (all data)	R_1 = 0.0758 wR_2 = 0.1919	R_1 = 0.0708, wR_2 = 0.1656	R_1 = 0.0561, wR_2 = 0.1053
Largest diff. peak/ hole/ e $Å^3$	0.726 and -0.479	0.953 and -0.326	4.042 and -0.877

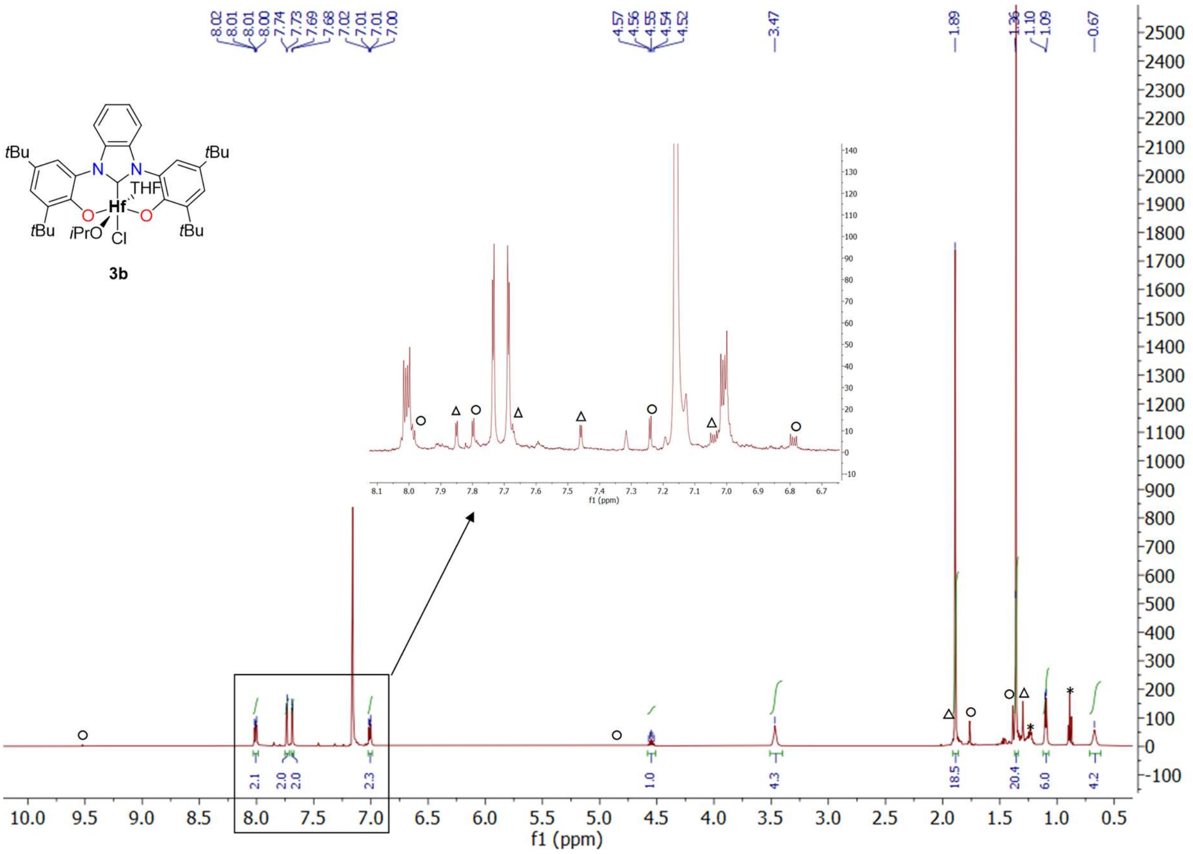

Figure S11. ^1H NMR spectrum of complex **3b** (major complex), **3b′** (○ = zwitterionic) and **3b′′** (Δ = homoleptic) in C_6D_6 (residual solvents: * = pentane).

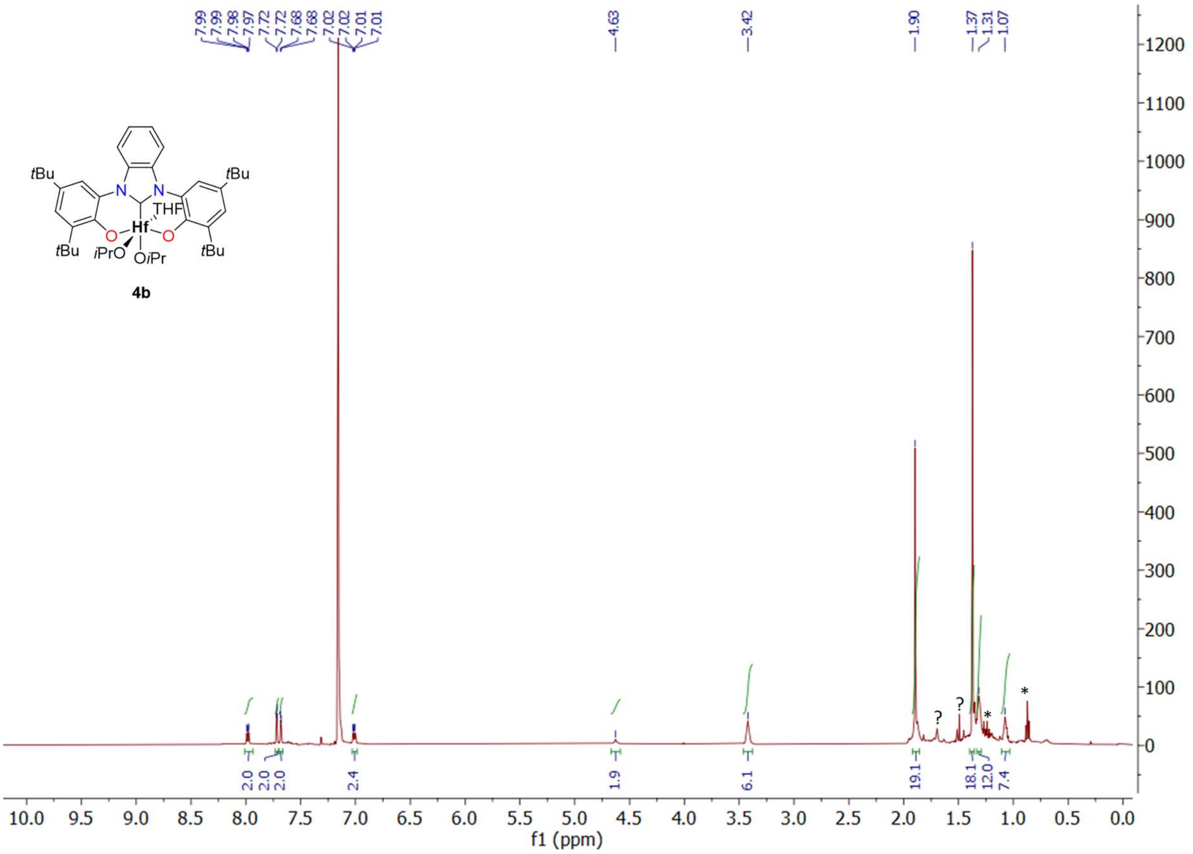

Figure S12. ^1H NMR spectrum of complex **4b** in C_6D_6 (residual solvents: * = pentane; ? = impurities).

Table S6. Interatomic distances, angles and torsion angles for 5-coordinate complexes **2a, 2b** and **2c**.

	(^{I}NHC)Ti(O*i*Pr)$_2$ (**2a**)	(^{Bz}NHC)TiCl(O*i*Pr)$_2$ (**2b**)	(^{Is}NHC)Ti(O*i*Pr)$_2$ (**2c**)[a]
		**Bond Length (Å)**	
Ti-$C_{carbene}$	2.183(3)	2.215(2)	2.212(4)
Ti-O*i*Pr[b]	1.817(2)/1.782(2)	1.7571(18)/1.8023(18)	1.792(5)/1.798(5)
		**Angle (°)**	
∠O_{Ar}-Ti-O_{Ar}	159.50(10)	138.19(8)	158.8(2)
∠Ti-O-C_{iPr}[b]	140.7(3)[c]/162.7(3)	158.0(3)[d]/145.8(2)[e]	139.9(5)/146.0(4)
∠N-$C_{carbene}$-N	104.7(2)	105.61(19)	108.5(5)
		**Dihedral Angle (°)**	
∠O_{Ar}-C_{Ar}-N-$C_{carbene}$	-12.88	-28.95	12.90
∠O_{Ar}-C_{Ar}-N-$C_{carbene}$	19.19	30.36	13.43

[a] Ref. [1]. [b] For **2a** and **2b**: apical\basal and for **2c**: equatorial /equatorial. [c] Disordered ∠Ti-O-C_{iPr}, other angle is at 146.4(3)°. [d] Disordered ∠Ti-O-C_{iPr}, other angle is at 136.4(4)°.

Table S7. Interatomic distances, angles and torsion angles for 6-coordinate complexes **3b** and **3c**.

	(^{Bz}NHC)HfCl(O*i*Pr)(THF) (**3b**)	(^{Is}NHC)HfCl(O*i*Pr)(THF) (**3c**)[a]
	**Bond Length (Å)**	
Hf-$C_{carbene}$	2.357(3)	2.333(3)
Hf-Cl	2.4393(8)	2.4614(9)
Hf-O*i*Pr	1.890(2)	2.014(3)
Hf-THF	2.318(2)	2.309(2)
	**Angle (°)**	
∠O_{Ar}-Hf-O_{Ar}	151.61(9)	154.71(8)
∠Hf-O-C_{iPr}	162.4(3)	171.1(3)
∠N-$C_{carbene}$-N	105.8(3)	108.7(2)
	**Dihedral Angle (°)**	
∠O_{Ar}-C_{Ar}-N-$C_{carbene}$	-27.82	7.93
∠O_{Ar}-C_{Ar}-N-$C_{carbene}$	26.10	7.65

[a] Ref. [2].

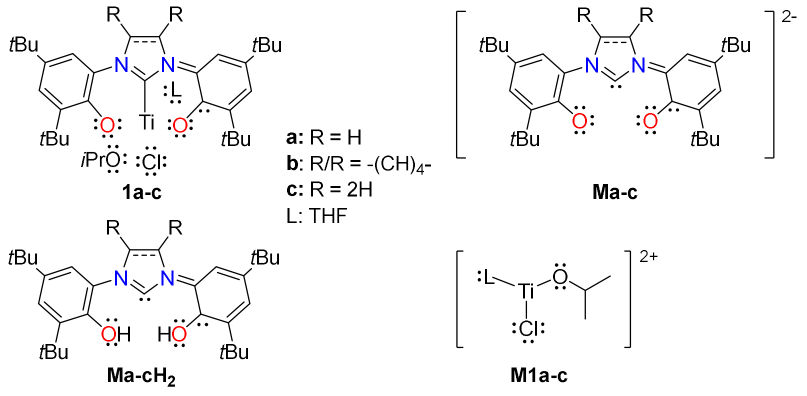

Scheme S2. Lewis structures used in the NBO analyses.

Cartesian Coordinates

103
1a

Ti	1.243417	9.190993	9.579172
Cl	2.096344	9.629431	7.448118
O	0.133423	7.714577	9.056988
N	-0.462171	10.122633	12.082064
C	-0.099684	9.106376	11.247785
O	1.734350	10.798519	10.468346
N	-0.794433	8.016481	11.681683
C	-1.364624	9.664242	13.040026
H	-1.760174	10.290756	13.826360
O	2.506672	8.153029	10.295545
C	-1.575670	8.345233	12.786699
H	-2.225067	7.635939	13.279909
O	-0.560360	10.452354	8.851156
C	-0.785224	6.745770	11.037977
C	-1.217152	5.617070	11.741436
H	-1.458432	5.714837	12.797776
C	-1.309450	4.381209	11.111936
C	-0.982188	4.326233	9.745252
H	-1.077513	3.369109	9.236704
C	-0.530292	5.418371	9.007527
C	-0.374287	6.663242	9.685882
C	-1.743223	3.107931	11.843054
C	-2.070360	3.369877	13.318803
H	-2.373694	2.430892	13.802360
H	-2.899550	4.083514	13.430880
H	-1.199819	3.759023	13.866102
C	-3.000372	2.527671	11.164639
H	-3.317587	1.604812	11.672506
H	-2.815449	2.284755	10.109936
H	-3.831140	3.246160	11.203840
C	-0.602897	2.071860	11.776203
H	-0.897458	1.143068	12.287001
H	0.304066	2.459894	12.260667
H	-0.347958	1.820883	10.738043
C	-0.213499	5.303979	7.508783
C	-0.528389	3.900521	6.969645
H	-0.314356	3.876494	5.892306
H	-1.586372	3.633498	7.105984
H	0.090654	3.126742	7.446193
C	-1.075702	6.312548	6.718216
H	-0.887494	6.195280	5.640987
H	-0.832456	7.343921	6.995598
H	-2.145834	6.134318	6.899001
C	1.279423	5.586368	7.238675
H	1.489819	5.451411	6.167390
H	1.915718	4.884215	7.796868
H	1.556294	6.610964	7.508011
C	0.012666	11.462781	11.981635
C	-0.657763	12.478010	12.671942
H	-1.573363	12.238992	13.207414
C	-0.183201	13.784563	12.657882
C	1.006984	14.032117	11.952324
H	1.406506	15.044062	11.963119
C	1.701631	13.060440	11.235218
C	1.162396	11.739535	11.202681
C	-0.897988	14.933508	13.374279
C	-2.172766	14.468221	14.089889
H	-2.652092	15.324275	14.584802
H	-1.954037	13.718159	14.863722
H	-2.901839	14.040496	13.386546
C	0.047692	15.550898	14.424163
H	-0.447379	16.386365	14.941066
H	0.966393	15.936812	13.963196
H	0.337109	14.802787	15.175347
C	-1.287922	16.011316	12.342643
H	-1.792020	16.853725	12.839191
H	-1.970717	15.597925	11.586874
H	-0.406524	16.405170	11.819553
C	3.023515	13.378652	10.521662
C	3.457406	14.834002	10.753713
H	4.417799	15.003154	10.247633
H	3.599347	15.057939	11.820865
H	2.735151	15.552238	10.339033
C	2.892958	13.168953	8.998867
H	3.845049	13.425385	8.511030
H	2.117601	13.829310	8.582474
H	2.652098	12.132636	8.739748
C	4.138142	12.465813	11.078754
H	5.091447	12.704377	10.584680
H	3.915246	11.407838	10.905549
H	4.264272	12.626718	12.159177
C	3.006416	6.824303	10.321210
H	2.266597	6.171843	9.827199
C	3.161494	6.384015	11.772473
H	3.529207	5.349250	11.821313
H	2.194847	6.433214	12.291492
H	3.874982	7.036101	12.295482
C	4.310664	6.782115	9.533915
H	4.695241	5.753523	9.483656
H	4.147085	7.149790	8.512776
H	5.066753	7.418039	10.015401
C	-0.442232	11.707874	8.123796
H	0.077357	11.501284	7.177078
H	0.172346	12.383727	8.728577
C	-1.873931	12.183974	7.922983
H	-1.974158	12.834938	7.045755
H	-2.223121	12.737723	8.807143
C	-2.632833	10.858483	7.786113
H	-3.709056	10.948368	7.979162
H	-2.495428	10.441220	6.778299
C	-1.932665	9.983686	8.817875
H	-2.365799	10.113385	9.824409
H	-1.919057	8.916947	8.568613

109
1b-THF

Ti	6.715626	13.853449	0.082313
Cl	8.873988	13.311508	-0.591338
N	3.628882	13.591906	-0.611720
C	4.597652	14.447604	-0.167873
O	6.934485	15.726498	-0.049011
N	4.204538	15.700526	-0.550300
C	3.047866	15.636043	-1.342201
O	6.730039	13.785100	1.870411
C	2.677822	14.277084	-1.380996
O	6.346986	14.034339	-2.228483
C	1.620448	13.838243	-2.182151
H	1.363285	12.784183	-2.252366
C	0.937857	14.797405	-2.927103
H	0.113825	14.484030	-3.567758
C	1.307517	16.151905	-2.889387
H	0.766176	16.873005	-3.501449
C	2.371508	16.591785	-2.104717
H	2.685258	17.632645	-2.117284
C	3.586910	12.203752	-0.291398
C	4.802502	11.507612	-0.092970
C	4.752770	10.125414	0.225125
C	3.494687	9.522939	0.335168
H	3.458132	8.463921	0.572766
C	2.285358	10.213705	0.184495
C	2.356019	11.574195	-0.118201
H	1.447441	12.169417	-0.187357
C	6.046007	9.342116	0.484302
C	6.744986	9.917409	1.736286
H	7.676581	9.364628	1.928207
H	6.097359	9.812146	2.618725
H	6.993749	10.976748	1.614078
C	6.994610	9.433981	-0.730277
H	7.878562	8.803494	-0.554946
H	7.338942	10.459791	-0.896335
H	6.495178	9.072816	-1.641323
C	5.768294	7.853648	0.743153
H	6.723133	7.337678	0.914137
H	5.276941	7.371065	-0.114329
H	5.144734	7.697436	1.635016
C	0.916134	9.549200	0.363115
C	0.181630	10.206037	1.549328
H	-0.808608	9.747539	1.689940

H	0.034731	11.282554	1.387007
H	0.754883	10.081463	2.478433
C	0.080593	9.738710	-0.918900
H	-0.905675	9.263989	-0.808547
H	0.585668	9.286952	-1.784203
H	-0.085020	10.802369	-1.139974
C	1.033905	8.044835	0.640935
H	0.029920	7.610787	0.748295
H	1.584208	7.844364	1.570783
H	1.540885	7.518247	-0.179906
C	4.873491	16.898622	-0.167532
C	6.273226	16.870950	0.042471
C	6.941892	18.085714	0.347758
C	6.165583	19.240167	0.497727
H	6.675800	20.168055	0.738195
C	4.769919	19.260145	0.376506
C	4.136634	18.063150	0.040150
H	3.051614	18.016499	-0.030945
C	8.468636	18.117592	0.508703
C	9.145885	17.604177	-0.780320
H	10.238039	17.690021	-0.683187
H	8.833222	18.202552	-1.648537
H	8.904923	16.552047	-0.965990
C	8.902092	17.233770	1.697487
H	9.990391	17.311268	1.837105
H	8.657961	16.181002	1.523860
H	8.416255	17.563656	2.627256
C	8.981753	19.541152	0.772210
H	10.076599	19.514391	0.859359
H	8.585445	19.957268	1.709630
H	8.729522	20.228762	-0.047914
C	3.929773	20.520386	0.608580
C	3.098601	20.826018	-0.653658
H	2.491721	21.731193	-0.503844
H	2.412658	20.002349	-0.895810
H	3.752710	20.989563	-1.521597
C	2.977942	20.283925	1.798662
H	2.358614	21.175294	1.978379
H	3.546314	20.068481	2.714086
H	2.303774	19.436420	1.614284
C	4.797853	21.746427	0.920574
H	4.153926	22.623975	1.072330
H	5.488590	21.976548	0.097116
H	5.388364	21.604540	1.836461
C	6.528158	14.611594	3.005864
C	6.443855	12.829004	-3.049440
H	5.427728	12.417080	-3.133961
H	7.084844	12.109097	-2.528892
C	7.017293	13.280421	-4.393028
H	6.590030	12.719926	-5.233589
H	8.106629	13.138156	-4.403300
C	6.683189	14.777246	-4.428383
H	7.334019	15.349506	-5.101204
H	5.638435	14.939335	-4.732198
C	6.875051	15.162323	-2.971299
H	7.938678	15.291433	-2.716397
H	6.318433	16.052712	-2.656706
O	5.956979	12.148133	-0.195923
H	6.588701	15.665005	2.678268
C	5.134850	14.343916	3.566054
H	4.935281	14.991549	4.431537
H	5.047623	13.294959	3.882193
H	4.374748	14.538653	2.797605
C	7.637220	14.336803	4.014568
H	8.620081	14.522816	3.563242
H	7.598417	13.288978	4.343771
H	7.525830	14.986764	4.893946

105

1c

C	10.665879	15.206281	4.516859
H	10.803291	15.674422	5.509847
C	10.778189	16.284144	3.443093
H	11.768912	16.754309	3.475227
H	10.012358	17.057149	3.598912
H	10.641324	15.844863	2.446470
C	9.321999	14.482190	4.495048
H	9.148392	14.018397	3.515833
H	8.506623	15.191190	4.697715
H	9.294829	13.693671	5.258723
C	14.851785	12.538353	5.791341
C	14.303446	11.357607	6.354935
C	15.033961	10.608314	7.288781
H	14.601501	9.698244	7.695359
C	16.303063	11.008013	7.697805
C	16.809413	12.204533	7.167637
H	17.787611	12.541569	7.503791
C	16.124155	12.990530	6.240883
C	17.138851	10.204849	8.699129
C	16.420498	8.933741	9.170736
H	16.202709	8.255008	8.333403
H	15.476551	9.166642	9.684582
H	17.058942	8.390299	9.881248
C	18.467840	9.790304	8.036017
H	18.283045	9.158108	7.156048
H	19.090675	9.223458	8.744114
H	19.042296	10.665729	7.705615
C	17.437062	11.076785	9.935480
H	18.046634	10.518105	10.661378
H	16.504432	11.382361	10.430144
H	17.986535	11.987496	9.663292
C	16.693663	14.327837	5.744904
C	16.898007	14.309720	4.216080
H	17.585740	13.501379	3.926612
H	17.346034	15.261765	3.894814
H	15.954358	14.183418	3.675656
C	15.722261	15.467770	6.123250
H	14.741126	15.328224	5.657074
H	16.132621	16.431043	5.785791
H	15.590697	15.515919	7.213916
C	18.051878	14.641384	6.390431
H	17.983370	14.710542	7.485642
H	18.407236	15.612070	6.017875
H	18.813284	13.889968	6.135277
C	10.267422	11.517518	2.820240
C	10.032257	10.838584	4.046278
C	8.806542	10.212061	4.286547
H	8.635629	9.724550	5.245242
C	7.795239	10.208381	3.323684
C	8.065497	10.828681	2.098741
H	7.299219	10.812437	1.329915
C	9.273772	11.474631	1.809621
C	9.530496	12.117645	0.437969
C	10.785104	11.492863	-0.211205
H	10.674567	10.402081	-0.300495
H	11.686143	11.716758	0.370116
H	10.922702	11.904920	-1.221721
C	9.740357	13.638723	0.586107
H	9.888704	14.091164	-0.405630
H	10.622933	13.862796	1.194185
H	8.858533	14.109738	1.044119
C	8.351840	11.898032	-0.522134
H	8.157120	10.830404	-0.699362
H	8.590236	12.359050	-1.490582
H	7.426632	12.363464	-0.153115
C	6.454966	9.534019	3.636781
C	5.804542	10.231949	4.848405
H	4.841119	9.760227	5.093197
H	5.624387	11.294525	4.633905
H	6.444613	10.173791	5.739400
C	5.476975	9.611553	2.456910
H	4.532940	9.118539	2.728528
H	5.873652	9.105930	1.565303
H	5.245426	10.651814	2.188687
C	6.688255	8.047017	3.971073
H	7.348212	7.926015	4.841082
H	7.151281	7.525327	3.121829
H	5.733957	7.550841	4.202486
C	12.224998	11.426156	5.020189
C	10.957781	9.787527	6.125786
H	10.654181	8.801541	5.753033
H	10.203429	10.133104	6.848030
C	12.370020	9.800398	6.707200
H	12.384314	9.962779	7.791750

H	12.924486	8.875586	6.487938
C	13.555685	10.004730	2.335719
H	12.530119	10.202907	2.010761
H	13.548408	9.208436	3.102369
C	14.521437	9.680486	1.207556
H	14.283031	10.292689	0.326182
H	14.491865	8.622635	0.917635
C	15.862177	10.107832	1.817648
H	16.238609	9.325840	2.493369
H	16.635259	10.310149	1.066224
C	15.486334	11.365097	2.598697
H	16.042806	11.483812	3.536721
H	15.580708	12.281530	2.001071
N	13.000856	10.941607	6.008652
N	11.060451	10.746552	5.005604
O	11.722067	14.282204	4.360169
O	14.176138	13.221025	4.882306
O	11.407465	12.163736	2.625499
O	14.072078	11.218278	2.930575
Cl	13.707625	14.145396	1.823880
Ti	12.757764	13.031767	3.644757

79

MaH_2

O	1.208844	7.357156	9.907029
N	-0.468952	10.141640	12.136881
C	-0.198422	9.168290	11.213834
O	0.392437	11.229105	9.673541
N	-0.889141	8.074757	11.660731
C	-1.315367	9.664500	13.133413
H	-1.677322	10.281303	13.944082
C	-1.569743	8.360961	12.840573
H	-2.153280	7.625982	13.377186
C	-0.862465	6.802495	11.013255
C	-1.901808	5.898213	11.249966
H	-2.741235	6.215984	11.864626
C	-1.881403	4.626418	10.689563
C	-0.780700	4.298864	9.881123
H	-0.751158	3.306061	9.437595
C	0.268423	5.172439	9.597142
C	0.220936	6.471124	10.168583
C	-2.997904	3.602290	10.911667
C	-4.106203	4.143164	11.824207
H	-4.881981	3.376415	11.957730
H	-4.589357	5.032763	11.395229
H	-3.721244	4.402651	12.821087
C	-3.626969	3.229001	9.554284
H	-4.425157	2.484774	9.693339
H	-2.882660	2.801662	8.869289
H	-4.060986	4.114510	9.069362
C	-2.407385	2.336747	11.565266
H	-3.194481	1.584459	11.722804
H	-1.957227	2.574735	12.539212
H	-1.629030	1.883660	10.937108
C	1.436754	4.753139	8.693431
C	1.287061	3.306868	8.197990
H	2.149513	3.057018	7.564631
H	0.380235	3.169579	7.591688
H	1.265136	2.586082	9.027979
C	1.494207	5.665819	7.449480
H	2.325283	5.354045	6.799593
H	1.643854	6.714590	7.724560
H	0.562489	5.585967	6.871317
C	2.763959	4.839019	9.477536
H	3.597865	4.531384	8.829311
H	2.741582	4.164124	10.345258
H	2.958530	5.856001	9.832826
C	0.023202	11.478753	12.044853
C	0.085229	12.260594	13.201932
H	-0.174755	11.805162	14.155052
C	0.498229	13.586532	13.144896
C	0.848566	14.095989	11.883916
H	1.171871	15.132910	11.824557
C	0.825206	13.350742	10.705542
C	0.413878	11.994509	10.788505
C	0.591847	14.481657	14.383691
C	0.167572	13.746952	15.661936
H	0.248025	14.425850	16.522271
H	0.809382	12.877458	15.864178
H	-0.875810	13.404054	15.606372
C	2.047059	14.959873	14.560345
H	2.131346	15.614294	15.440655
H	2.394444	15.524882	13.685209
H	2.723805	14.105235	14.699168
C	-0.328326	15.705306	14.200480
H	-0.264619	16.366932	15.077096
H	-1.374748	15.391742	14.079884
H	-0.048951	16.291305	13.314871
C	1.235845	13.967225	9.360578
C	1.629855	15.444923	9.504575
H	1.906459	15.837000	8.516069
H	2.496152	15.578912	10.168176
H	0.800235	16.058316	9.884545
C	0.055355	13.897503	8.368061
H	0.350861	14.347549	7.408700
H	-0.806079	14.460015	8.755911
H	-0.257771	12.864835	8.184216
C	2.456709	13.215211	8.788111
H	2.754000	13.666879	7.830058
H	2.235789	12.156683	8.618574
H	3.310229	13.287761	9.477291
H	0.915619	8.255913	10.283770
H	0.214218	10.271496	9.968341

85

MbH_2

N	3.664245	13.627836	-0.445877
C	4.575354	14.509047	0.077096
O	7.018231	15.781503	-0.161056
N	4.219010	15.737659	-0.404086
C	3.129924	15.643643	-1.282759
C	2.773780	14.281396	-1.318226
C	1.787183	13.825254	-2.197781
H	1.545104	12.769465	-2.284552
C	1.154971	14.769632	-3.004061
H	0.386614	14.438671	-3.702517
C	1.502660	16.129281	-2.953382
H	0.997021	16.837707	-3.609206
C	2.503785	16.586440	-2.099864
H	2.809614	17.630354	-2.092018
C	3.651869	12.235973	-0.119881
C	4.854929	11.590220	0.258664
C	4.845592	10.192341	0.471112
C	3.621614	9.521476	0.363325
H	3.615065	8.448830	0.530717
C	2.407638	10.160525	0.087842
C	2.447687	11.534466	-0.149072
H	1.527349	12.084884	-0.330022
C	6.137301	9.442300	0.828134
C	6.681771	9.949426	2.180960
H	7.600263	9.401918	2.440013
H	5.946420	9.777015	2.979869
H	6.913365	11.018698	2.146303
C	7.191475	9.651756	-0.279935
H	8.112336	9.107159	-0.023664
H	7.440262	10.710218	-0.407568
H	6.822587	9.261948	-1.239560
C	5.903502	7.929818	0.960787
H	6.859323	7.441683	1.196679
H	5.525447	7.487690	0.027815
H	5.200355	7.688934	1.770825
C	1.064083	9.424514	0.053717
C	0.150355	9.984495	1.162498
H	-0.824633	9.474761	1.151432
H	-0.029773	11.060244	1.031349
H	0.605209	9.838297	2.152011
C	0.389970	9.639809	-1.316127
H	-0.573676	9.110783	-1.357293
H	1.026173	9.261601	-2.128501
H	0.192364	10.703811	-1.507296
C	1.225355	7.914981	0.276579
H	0.240232	7.429583	0.234719
H	1.664049	7.693122	1.259455
H	1.858407	7.455116	-0.495420

C	4.912703	16.944930	-0.084923
C	6.322971	16.939840	-0.010608
C	7.003838	18.157082	0.211225
C	6.229776	19.312104	0.383768
H	6.751811	20.248266	0.556283
C	4.830260	19.324554	0.369993
C	4.183674	18.110235	0.135790
H	3.096148	18.048815	0.136161
C	8.537231	18.205770	0.270008
C	9.130860	17.700138	-1.062293
H	10.229180	17.744108	-1.019105
H	8.798263	18.334309	-1.896593
H	8.835137	16.667128	-1.272374
C	9.043666	17.335814	1.440657
H	10.141902	17.379649	1.487072
H	8.743886	16.289295	1.325455
H	8.647755	17.708844	2.395977
C	9.057143	19.633523	0.494915
H	10.155484	19.612350	0.519223
H	8.712827	20.053054	1.450946
H	8.755911	20.314716	-0.313784
C	4.002841	20.591887	0.607694
C	3.079902	20.837474	-0.602929
H	2.477928	21.745095	-0.448366
H	2.385381	20.000402	-0.759274
H	3.668211	20.966946	-1.522255
C	3.142355	20.407130	1.873764
H	2.532460	21.304048	2.057857
H	3.777187	20.234719	2.753908
H	2.461853	19.550252	1.777472
C	4.884959	21.832587	0.798797
H	4.249116	22.715035	0.956085
H	5.512778	22.025419	-0.082602
H	5.540936	21.735024	1.675147
O	5.997211	12.298478	0.427662
H	6.362369	15.040303	-0.040504
H	5.718570	13.274136	0.480040

81

McH_2

C	15.099910	12.027125	5.468331
C	14.331493	11.262639	6.381251
C	14.856691	10.915508	7.633251
H	14.245630	10.333054	8.317730
C	16.136701	11.309483	8.012185
C	16.878726	12.063510	7.092645
H	17.880448	12.378111	7.376230
C	16.405444	12.435492	5.832496
C	16.743430	10.951816	9.372659
C	15.782927	10.126200	10.238649
H	15.520539	9.172125	9.758860
H	14.854530	10.675019	10.453113
H	16.260329	9.893216	11.200691
C	18.029216	10.128176	9.158186
H	17.808816	9.194795	8.621368
H	18.486612	9.870992	10.125273
H	18.771025	10.685636	8.571171
C	17.088998	12.246630	10.135155
H	17.537313	12.009425	11.111542
H	16.186642	12.850037	10.306764
H	17.804296	12.864241	9.576147
C	17.269197	13.260778	4.866859
C	17.514623	12.464148	3.567411
H	18.042916	11.525200	3.787110
H	18.141712	13.054698	2.882982
H	16.576328	12.224101	3.057351
C	16.568824	14.598641	4.545870
H	15.594016	14.441360	4.073484
H	17.195361	15.190887	3.862413
H	16.421019	15.184214	5.464609
C	18.642956	13.593301	5.468668
H	18.557779	14.193113	6.386141
H	19.217372	14.181950	4.739913
H	19.224370	12.688245	5.695906
C	10.084231	11.840553	3.153271
C	10.105015	10.714444	4.013357
C	9.070359	9.770248	3.970361
H	9.112029	8.912725	4.636743
C	8.002958	9.910387	3.088169
C	8.003034	11.032446	2.248126
H	7.174765	11.153843	1.553819
C	9.006314	12.003954	2.250437
C	8.947602	13.214139	1.306142
C	10.178491	13.215369	0.374326
H	10.198025	12.301363	-0.236446
H	11.113614	13.275378	0.940046
H	10.127591	14.078063	-0.306550
C	8.900729	14.520945	2.126765
H	8.849286	15.384445	1.446932
H	9.785983	14.631735	2.761036
H	8.007385	14.539533	2.767525
C	7.696335	13.185292	0.415277
H	7.669761	12.295893	-0.230494
H	7.704374	14.069069	-0.237739
H	6.768772	13.216871	1.004708
C	6.856525	8.897329	3.009834
C	5.523991	9.607393	3.322405
H	4.686265	8.896867	3.259487
H	5.327588	10.424510	2.615772
H	5.538784	10.035155	4.334702
C	6.795694	8.302682	1.588510
H	5.969435	7.580452	1.509328
H	7.732879	7.783785	1.343064
H	6.636171	9.082759	0.832445
C	7.031014	7.743725	4.006206
H	7.052692	8.103405	5.045181
H	7.953423	7.176625	3.814713
H	6.187341	7.045479	3.914605
C	12.312303	11.228326	4.973848
C	11.072506	9.518567	6.018306
H	11.093551	8.513014	5.573583
H	10.138031	9.629094	6.582150
C	12.317683	9.811772	6.856633
H	12.070939	10.245705	7.836502
H	12.946660	8.927471	7.017152
N	13.037178	10.811690	6.030912
N	11.152219	10.542803	4.948878
O	14.606746	12.359126	4.249968
O	11.073503	12.766625	3.194105
H	13.646332	12.041920	4.223177
H	11.772554	12.424342	3.840505

77

Ma

O	0.151131	1.881788	0.347272
N	0.397537	-1.001304	2.980068
C	0.000000	0.000000	2.143677
O	0.145917	-1.868530	0.321081
N	-0.378929	1.024348	2.959728
C	0.251322	-0.608715	4.309253
H	0.473500	-1.257119	5.144425
C	-0.231721	0.662113	4.296359
H	-0.454199	1.331570	5.115140
C	-0.784215	2.311341	2.503063
C	-1.491909	3.154093	3.365801
H	-1.805320	2.772827	4.335356
C	-1.817986	4.449791	2.982416
C	-1.381850	4.877863	1.715732
H	-1.608263	5.899392	1.417389
C	-0.690876	4.069833	0.815255
C	-0.425827	2.721247	1.196397
C	-2.620233	5.401744	3.873545
C	-2.993528	4.761757	5.216877
H	-3.568259	5.477993	5.820387
H	-2.101318	4.481427	5.795468
H	-3.616615	3.865985	5.082098
C	-1.786650	6.667725	4.156035
H	-2.355267	7.367280	4.786528
H	-1.519399	7.191561	3.228793
H	-0.854295	6.411839	4.678698
C	-3.921759	5.798976	3.147945
H	-4.510051	6.492744	3.766760
H	-4.538004	4.912707	2.941480
H	-3.713229	6.293621	2.190030

C	-0.215678	4.601533	-0.545319
C	-0.535936	6.094541	-0.712288
H	-0.152383	6.434622	-1.684028
H	-0.061805	6.708336	0.067247
H	-1.618149	6.288646	-0.698661
C	1.315699	4.436415	-0.660292
H	1.663267	4.864062	-1.612086
H	1.602567	3.379520	-0.637495
H	1.825960	4.965333	0.157945
C	-0.899474	3.842705	-1.702091
H	-0.579445	4.273287	-2.662461
H	-1.993121	3.939486	-1.638549
H	-0.634475	2.780276	-1.706357
C	0.896130	-2.265931	2.551492
C	1.566773	-3.081700	3.468796
H	1.764321	-2.703074	4.468697
C	2.003413	-4.351480	3.108947
C	1.715562	-4.788075	1.804278
H	2.021972	-5.793279	1.522420
C	1.067104	-4.008140	0.849220
C	0.687970	-2.681529	1.213886
C	2.765922	-5.268515	4.069073
C	2.980773	-4.620930	5.443163
H	3.533386	-5.311833	6.094924
H	2.026107	-4.390859	5.938070
H	3.567104	-3.693591	5.369366
C	1.972240	-6.575293	4.269414
H	2.512531	-7.250998	4.948969
H	1.818767	-7.104487	3.319766
H	0.983641	-6.367998	4.702325
C	4.147239	-5.597844	3.467826
H	4.707539	-6.267699	4.136919
H	4.737515	-4.681831	3.323660
H	4.053714	-6.094172	2.492880
C	0.736970	-4.555350	-0.546978
C	1.161842	-6.024205	-0.695649
H	0.880660	-6.375426	-1.697978
H	0.663355	-6.674970	0.037238
H	2.249358	-6.152670	-0.594260
C	1.463384	-3.747467	-1.642228
H	1.228022	-4.173957	-2.628600
H	2.553751	-3.805267	-1.506616
H	1.163037	-2.694469	-1.652108
C	-0.789128	-4.486450	-0.775672
H	-1.032302	-4.896374	-1.766955
H	-1.156389	-3.455945	-0.729078
H	-1.319604	-5.083743	-0.020060

83
Mb

N	0.325552	1.096948	2.961216
C	0.000000	0.000000	2.213917
O	0.158777	-1.858304	0.308141
N	0.335246	-1.089663	2.969461
C	0.985419	-0.693398	4.148191
C	0.978500	0.715545	4.141875
C	1.649029	1.446290	5.126190
H	1.687021	2.532487	5.097262
C	2.309800	0.728646	6.120797
H	2.848581	1.271094	6.897415
C	2.317739	-0.675864	6.126406
H	2.862837	-1.206176	6.906976
C	1.664362	-1.408830	5.137902
H	1.715217	-2.494659	5.118508
C	0.000584	2.434811	2.592628
C	-0.054366	2.775109	1.220465
C	-0.378026	4.109375	0.861153
C	-0.636213	5.021215	1.890591
H	-0.877936	6.043298	1.614500
C	-0.627957	4.682855	3.249922
C	-0.317154	3.363420	3.581648
H	-0.354585	3.034310	4.618408
C	-0.484285	4.510952	-0.615545
C	-1.644501	3.729378	-1.271322
H	-1.726213	4.007432	-2.332585
H	-2.597174	3.974226	-0.779732
H	-1.492389	2.646667	-1.211196
C	0.833623	4.209778	-1.361131
H	0.762669	4.575923	-2.395795
H	1.040481	3.135354	-1.396476
H	1.679054	4.720706	-0.877315
C	-0.776095	6.010262	-0.778583
H	-0.834230	6.247354	-1.849837
H	0.016925	6.634239	-0.341135
H	-1.735366	6.297470	-0.324755
C	-0.967410	5.681795	4.361290
C	-2.230910	5.205643	5.106191
H	-2.487729	5.906441	5.914577
H	-2.085508	4.213088	5.553975
H	-3.086262	5.142376	4.419354
C	0.207618	5.763985	5.356356
H	-0.018706	6.480349	6.160010
H	1.125722	6.093127	4.849714
H	0.409144	4.790735	5.825231
C	-1.231533	7.090458	3.813496
H	-1.456914	7.772459	4.645353
H	-2.090428	7.106392	3.128162
H	-0.357462	7.489714	3.279925
C	0.035539	-2.434581	2.607779
C	-0.008302	-2.787656	1.237545
C	-0.231077	-4.146004	0.889367
C	-0.469764	-5.056272	1.924910
H	-0.647219	-6.093932	1.658649
C	-0.513620	-4.699792	3.279152
C	-0.255802	-3.366962	3.601762
H	-0.312967	-3.030687	4.635276
C	-0.211170	-4.588659	-0.580847
C	1.147886	-4.232032	-1.220865
H	1.180089	-4.608819	-2.253628
H	1.974103	-4.696598	-0.663051
H	1.302442	-3.148208	-1.251727
C	-1.341906	-3.891844	-1.367052
H	-1.352358	-4.260963	-2.403167
H	-1.199684	-2.806958	-1.396413
H	-2.321933	-4.110831	-0.918806
C	-0.410473	-6.105337	-0.719460
H	-0.368196	-6.374038	-1.783890
H	-1.387440	-6.431489	-0.334586
H	0.375222	-6.673544	-0.200945
C	-0.841206	-5.697666	4.394878
C	0.314859	-5.729154	5.414693
H	0.095920	-6.444410	6.221268
H	0.473449	-4.745029	5.877225
H	1.253977	-6.032349	4.930904
C	-2.135370	-5.255535	5.107514
H	-2.385488	-5.955161	5.918913
H	-2.977837	-5.229514	4.402505
H	-2.032800	-4.253487	5.545854
C	-1.047156	-7.120212	3.858196
H	-1.272422	-7.799052	4.692578
H	-0.147806	-7.498762	3.352206
H	-1.887374	-7.170962	3.151696
O	0.184639	1.853980	0.299552

79
Mc

C	0.628391	2.717952	1.205846
C	0.738743	2.326481	2.564907
C	1.224869	3.223484	3.527315
H	1.316714	2.897369	4.559675
C	1.595718	4.519686	3.180887
C	1.433315	4.905623	1.841556
H	1.691626	5.925928	1.566389
C	0.950172	4.056649	0.845535
C	2.150199	5.519991	4.199810
C	2.250864	4.921320	5.608930
H	2.920682	4.049559	5.633752
H	1.266679	4.615095	5.992235
H	2.656977	5.671913	6.301353
C	3.560642	5.963577	3.762020
H	4.242926	5.102948	3.717611
H	3.972296	6.694543	4.473968
H	3.544662	6.430758	2.768531
C	1.226124	6.752722	4.264779

H	1.615990	7.487331	4.985183
H	0.213603	6.463766	4.579699
H	1.145338	7.247737	3.288134
C	0.725667	4.550324	-0.591340
C	1.583473	3.749413	-1.592389
H	2.652027	3.841823	-1.347771
H	1.435822	4.151708	-2.605645
H	1.313787	2.688540	-1.609717
C	-0.768207	4.400405	-0.954765
H	-1.088437	3.354208	-0.906341
H	-0.939113	4.768346	-1.977301
H	-1.392705	4.992282	-0.270028
C	1.095132	6.033246	-0.746900
H	0.495753	6.680101	-0.090176
H	0.901142	6.341365	-1.783597
H	2.159598	6.217111	-0.540559
C	-0.270221	-2.766428	1.203511
C	-0.578611	-2.375219	2.534403
C	-1.087256	-3.303289	3.446873
H	-1.346724	-2.975450	4.452444
C	-1.277087	-4.639681	3.089280
C	-0.910956	-5.021353	1.793683
H	-1.028149	-6.062083	1.507876
C	-0.402410	-4.130675	0.840349
C	0.009967	-4.606441	-0.561220
C	1.495362	-4.268970	-0.817219
H	2.135213	-4.717940	-0.043259
H	1.658969	-3.185997	-0.835548
H	1.804156	-4.674871	-1.791773
C	-0.857919	-3.924980	-1.639204
H	-0.575832	-4.299821	-2.634278
H	-0.718711	-2.838923	-1.636273
H	-1.922296	-4.151966	-1.481654
C	-0.155336	-6.125525	-0.717160
H	0.457990	-6.683825	0.004842
H	0.167873	-6.417613	-1.725864
H	-1.202178	-6.440881	-0.601039
C	-1.863451	-5.620273	4.110917
C	-3.272908	-5.146902	4.520233
H	-3.711634	-5.835842	5.257466
H	-3.938025	-5.106562	3.646433
H	-3.247702	-4.144329	4.968741
C	-1.980117	-7.043166	3.548476
H	-2.406036	-7.707440	4.313574
H	-0.999957	-7.449194	3.261427
H	-2.639077	-7.080050	2.669776
C	-0.960637	-5.669410	5.359862
H	-0.874436	-4.683287	5.836551
H	0.051724	-6.005304	5.095157
H	-1.372301	-6.366959	6.104432
C	0.000000	0.000000	2.180284
C	-0.320583	-0.713736	4.394126
H	0.265103	-1.449179	4.959263
H	-1.350866	-0.720056	4.779593
C	0.297536	0.682833	4.406343
H	-0.301279	1.410425	4.967457
H	1.319746	0.687724	4.813587
N	0.327163	1.039891	2.971103
N	-0.317732	-1.056108	2.956123
O	0.206365	1.857328	0.294745
O	0.150504	-1.867249	0.326192

26
M1a

Ti	0.000000	0.000000	0.000000
Cl	0.752617	0.091742	-2.210487
O	-1.773925	0.048498	-0.192893
O	2.260706	0.000000	0.513672
C	-2.867602	0.921342	-0.433578
H	-2.506069	1.958359	-0.328829
C	-3.942892	0.665207	0.616285
H	-4.794930	1.343004	0.464759
H	-3.540279	0.834469	1.623985
H	-4.301845	-0.371452	0.550620
C	-3.353706	0.706214	-1.861898
H	-4.163857	1.409586	-2.101371
H	-2.528593	0.861773	-2.568765
H	-3.729030	-0.319283	-1.985938
C	3.257925	-0.805701	-0.176067
H	3.272515	-0.493998	-1.230371
H	2.936488	-1.851377	-0.117049
C	4.566528	-0.517334	0.545848
H	5.438916	-0.676422	-0.099875
H	4.668258	-1.162688	1.430991
C	4.380841	0.945421	0.967113
H	5.040976	1.251620	1.788130
H	4.557007	1.614878	0.113023
C	2.911188	0.976866	1.366071
H	2.766492	0.669189	2.415802
H	2.421869	1.943859	1.205629

26
M1b

Ti	0.000000	0.000000	0.000000
Cl	0.922241	-0.030644	-2.134142
O	-1.774068	0.003143	-0.234197
O	2.251599	0.000000	0.662344
C	-2.924238	-0.776637	0.052431
C	3.076572	1.162339	0.338967
H	3.040339	1.832579	1.210061
H	2.634080	1.667974	-0.526272
C	4.478939	0.618976	0.063362
H	5.262591	1.300380	0.416675
H	4.617151	0.466216	-1.015786
C	4.476811	-0.732784	0.788689
H	5.222350	-1.435251	0.395675
H	4.655777	-0.600656	1.866000
C	3.052972	-1.202801	0.543826
H	2.925204	-1.618862	-0.467863
H	2.676295	-1.922626	1.279727
H	-2.590327	-1.796744	0.314238
C	-3.644891	-0.166111	1.250216
H	-4.526944	-0.765320	1.517138
H	-3.970559	0.857191	1.016426
H	-2.970972	-0.125955	2.116480
C	-3.795782	-0.840529	-1.196305
H	-3.231657	-1.266647	-2.035649
H	-4.128723	0.168354	-1.477593
H	-4.681253	-1.465924	-1.014685

26
M1c

C	-3.072158	-0.367789	-0.540031
H	-3.737054	0.480987	-0.291932
C	-3.157744	-0.640321	-2.038623
H	-2.830514	0.239367	-2.606681
H	-4.191685	-0.882647	-2.322448
H	-2.508478	-1.482667	-2.310402
C	-3.473348	-1.572351	0.307832
H	-2.840602	-2.437851	0.074063
H	-4.521560	-1.840326	0.112802
H	-3.365507	-1.346247	1.376919
C	2.976021	-1.093750	1.208394
H	2.354397	-1.988274	1.108056
H	3.099889	-0.853479	2.280223
C	4.318028	-1.177591	0.499487
H	4.204413	-1.721376	-0.449112
H	5.083271	-1.681916	1.102841
C	4.623261	0.303279	0.242037
H	5.025135	0.772209	1.152209
H	5.343444	0.462420	-0.569896
C	3.247652	0.871796	-0.099276
H	3.089250	1.893007	0.269074
H	3.026355	0.834223	-1.174232
O	-1.760501	0.043132	-0.216454
O	2.282182	0.000000	0.563792
Cl	0.775640	-0.111322	-2.200907
Ti	0.000000	0.000000	0.000000

References:

1. Romain, C.; Brelot, L.; Bellemin-Laponnaz, S.; Dagorne, S., Synthesis and Structural Characterization of a Novel Family of Titanium Complexes Bearing a Tridentate Bis-phenolate-*N*-heterocyclic Carbene Dianionic Ligand and Their Use in the Controlled ROP of *rac*-Lactide. *Organometallics* **2010,** 29, (5), 1191-1198.
2. Lalrempuia, R.; Underhaug, J.; Törnroos, K. W.; Le Roux, E., Anionic hafnium species: an active catalytic intermediate for the coupling of epoxides with CO_2? *Chem. Commun.* **2019,** 55, (50), 7227-7230.
